# Metal–Metal Interactions in Hexagonal Perovskites Containing Bimetallic Face-Sharing Bioctahedra

**DOI:** 10.1021/acs.inorgchem.6c03181

**Published:** 2026-09-09

**Authors:** David Liu, Cierra J. Foster, Maxim Avdeev, Patrick M. Woodward

**Affiliations:** † Department of Chemistry and Biochemistry, 2647 The Ohio State University, 100 W. 18th Avenue, Columbus, Ohio 43210, United States; ‡ Australian Centre for Neutron Scattering, 5419 Australian Nuclear Science and Technology Organisation, Lucas Heights, NSW 2234, Australia; § School of Chemistry, The University of Sydney, Sydney, NSW 2006, Australia

## Abstract

Recent studies have revealed metal-to-metal and intervalence-charge transfer transitions in cation ordered 6H perovskites with Cs_3_M^+^M^2+^M^3+^Cl_9_ stoichiometry. The structure and properties of two new members of this family, Cs_3_NaMnVCl_9_ and Cs_3_NaMnCrCl_9_, are reported here. In both compounds Na^+^ ions occupy sites in octahedra that share only corners, electronically isolating face-sharing M_2_Cl_9_ bioctahedral clusters. Analysis of synchrotron X-ray and neutron powder diffraction patterns reveals no sign of long-range ordering of the Mn^2+^ and V^3+^/Cr^3+^ ions, suggesting a random orientation of bimetallic Mn^2+^M^3+^Cl_9_^4–^ clusters. Magnetometry studies reveal paramagnetic behavior in both compounds, with negative Weiss temperatures and effective moments that closely align with expected values based on spin-only moments. No sign of long-range magnetic order is observed in either compound down to at least 2 K. The optical absorption spectra feature d-to-d transitions, ligand-to-metal charge transfer (LMCT), and metal-to-metal charge transfer (MMCT) transitions. Cs_3_NaMnVCl_9_ exhibits a Mn^2+^ to V^3+^ MMCT transition at ∼1390 nm (∼0.9 eV), whereas the comparable MMCT transition in Cs_3_NaMnCrCl_9_ falls at a much higher energy and cannot be resolved due to overlap with the LMCT transition. The striking difference in MMCT energy can be attributed to differences in the electron configuration of V^3+^ (t_2g_^2^e_g_^0^) and Cr^3+^ (t_2g_^3^e_g_^0^).

## Graphical abstract

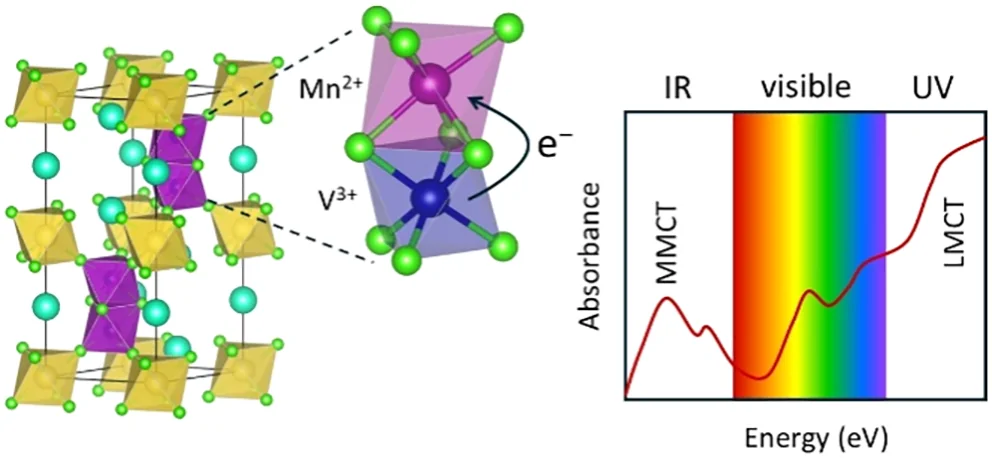

## Introduction

The vibrant blue color, durability, and rarity of sapphire have long made it a symbol of wealth and royalty. However, it was not until the 20th century that scientists realized that Fe^2+^ and Ti^4+^ defects in α-Al_2_O_3_ were responsible for the striking coloration. The overlap of metal orbitals through the shared faces and edges of the octahedra result in a broad Fe^2+^ → Ti^4+^ metal-to-metal charge transfer (MMCT) band at ∼600 nm that is responsible for its deep blue color.^1,2^ Such heteronuclear mixed valent pairings are present in a variety of gemstones, including dravite tourmaline, elbaite, and andalusite.^3–5^ However, these are often limited to Fe^2+^ → Fe^3+^, Mn^2+^ → Ti^4+^, and Fe^2+^ → Ti^4+^ MMCT. Identifying other structure types that can expand the variety of heteronuclear mixed valent pairings and facilitate MMCT transitions can enhance our fundamental understanding of such transitions.

Recent discoveries in the 6H family of perovskites present new opportunities to explore the interactions between various transition metal ions. Several compositions with the formula Cs_3_M^+^M^2+^M^3+^Cl_9_ have been shown to adopt the 6H hexagonal perovskite structure.^6–8^ The crystal structure consists of face-sharing bioctahedra held together by a layer of octahedra that exclusively share corners with M_2_X_9_ bioctahedra. The M^+^ cations, typically Na^+^ or Ag^+^, show a strong preference for sites in the layer of corner-sharing octahedra, while the M^2+^ and M^3+^ cations preferentially occupy sites in the M_2_X_9_ bioctahedra.

The bioctahedron creates an environment that is similar to the environment found in sapphire (corundum). It brings the M^2+^ and M^3+^ ions into close proximity through a shared face, facilitating optical transitions involving excitation of an electron from one metal to the other (i.e., MMCT). As a result, compounds such as Cs_3_NaMnFeCl_9_ and Cs_3_NaFe_2_Cl_9_ appear almost black, a sharp contrast from the orange color arising from ligand-to-metal charge transfer (LMCT) excitations in chloride perovskites like CsFeCl_3_ and Cs_2_NaFeCl_6_.^9,10^ In Cs_3_NaFe_2_Cl_9_ rapid hopping of an electron between two iron centers occupying the same bioctahedron leads to temperature dependent mixed valency and unexpectedly strong ferromagnetic coupling between iron centers.^6^ The intriguing properties found in these studies motivate a search for new Cs_3_M^+^M^2+^M^3+^Cl_9_ compounds with previously unexplored combinations of transition metal cations.

Here, we introduce two such compounds, Cs_3_NaMnCrCl_9_ and Cs_3_NaMnVCl_9_, which contain Mn^2+^–Cr^3+^ and Mn^2+^–V^3+^ pairings in the M_2_Cl_9_ clusters, respectively. This paper reports the synthesis and characterization of the structural, optical, and magnetic properties of these compounds. Interestingly, we find that the difference in the electron configuration of the trivalent cation (3d^2^ for V^3+^ vs 3d^3^ for Cr^3+^) leads to significant differences in the energies of charge transfer transitions. The results provide fundamental insight that can help to understand the optical and magnetic properties of materials containing face-sharing octahedra.

## Experimental Section

CsMnCl_3_ was first prepared via solution precipitation methods as a precursor to the synthesis of both Cs_3_NaMnVCl_9_ and Cs_3_NaMnCrCl_9_. A stoichiometric amount of MnCl_2_·4H_2_O (Sigma-Aldrich, 99.9%) was added to a round-bottom flask containing 10 mL of hydrochloric acid (Fisher Scientific, 37%) per gram of desired product. The solution was heated to 70 °C and stirred for 30 min to dissolve all solids. Afterward, a stoichiometric amount of CsCl (Alfa Aesar, 99.9%) was added to initiate precipitation of the pale pink CsMnCl_3_ product. The solution was then cooled, and the precipitate collected via vacuum filtration and washed with ethanol (Fisher Scientific, 99.9%).

Cs_3_NaMnVCl_9_ and Cs_3_NaMnCrCl_9_ were prepared through a conventional solid-state method. In the case of Cs_3_NaMnCrCl_9_, stoichiometric amounts of CsCl, NaCl (Fisher Scientific, 99.9%), CsMnCl_3_, and CrCl_3_ (Fisher Scientific, batch #122804) were ground together in an agate mortar and pestle. The powder was then pressed into a 13 mm diameter pellet and sealed in an evacuated quartz ampule. For Cs_3_NaMnVCl_9_, stoichiometric amounts of CsCl, NaCl, CsMnCl_3_, and VCl_3_ (Sigma-Aldrich, 98%) were ground together in an Ar-filled glovebox, transferred to an alumina crucible and sealed in an evacuated quartz ampule. The ampules containing Cs_3_NaMnVCl_9_ and Cs_3_NaMnCrCl_9_ were then heated at 550 and 500 °C, respectively, for a duration of 12 h and cooled at a rate of 100 °C/h. For both compounds, the products were ground again, sealed, and reheated for at least two cycles to increase purity.

Neutron powder diffraction (NPD) data were collected at the Australian Centre for Neutron Scattering on the Echidna beamline employing a wavelength of λ = 1.622 Å. Synchrotron X-ray powder diffraction (XRPD) data were collected at the Canadian Light Source on the BXDS-WHE beamline. The data for Cs_3_NaMnVCl_9_ and Cs_3_NaMnCrCl_9_ were collected at 300 K using 55.32 keV X-rays (λ = 0.2241 Å) and 33.34 keV X-rays (λ = 0.3719 Å), respectively, with a Varex area detector. Samples were packed into 0.8 mm Kapton capillaries and sealed with clay. Rietveld refinements of the synchrotron PXRD and NPD data were carried out using the TOPAS-Academic (Version 6) software package to determine the crystal structure.^11^ Images of the crystal structures were generated with Vesta 3.^12^

Diffuse reflectance spectroscopy (DRS) was collected on a PerkinElmer Lambda 950 spectrometer using a 60 mm InGaAs integrating sphere. Calibration was conducted using dried BaSO_4_ and data was collected between 250–2500 nm. The spectra were transformed to pseudoabsorbance using the Kubelka–Munk function: *F*(*R*) = α = (1 – *R*)^2^/(2*R*), where *R* is the reflectance and α is the optical absorption coefficient.^13^

Direct current magnetic susceptibility measurements were collected from 300 to 2 K with an applied field of 1000 Oe using a Quantum Design superconducting quantum interference device MPMS-3. The samples were prepared by loading 50–100 mg of the powder sample into a gel capsule, which was then mounted in a plastic straw. The diamagnetic contributions were corrected using the constants given by Pascal.^14^

## Results

### Structure

Powders of the purple Cs_3_NaMnCrCl_9_ and the reddish Cs_3_NaMnVCl_9_ were prepared through a conventional solid-state synthetic route as described above. Both compounds were studied using synchrotron X-ray powder diffraction (XRPD) data (Figure 1). Analysis of the powder diffraction data finds that both compounds adopt the 6H perovskite structure. This structure consists of layers of face-sharing bioctahedral M_2_Cl_9_ clusters separated by layers of octahedra that share corners with M_2_Cl_9_ clusters (Figure 2). Rietveld refinements indicate that the M(1) site, located in the corner-sharing octahedral layers, is exclusively occupied by Na^+^, while Mn^2+^ and V^3+^/Cr^3+^ occupy the octahedral sites within the face-sharing bioctahedra. In addition to the target phases described below, both samples contained small amounts of an unidentified secondary phase(s) as shown in the Supporting Information (Figures S1 and S3). The unindexed reflections in both compounds are inconsistent with the binary precursors, as well as any ternary and quaternary combinations of precursor ions that are reported in the Inorganic Crystal Structure Database.

**Figure 1:**
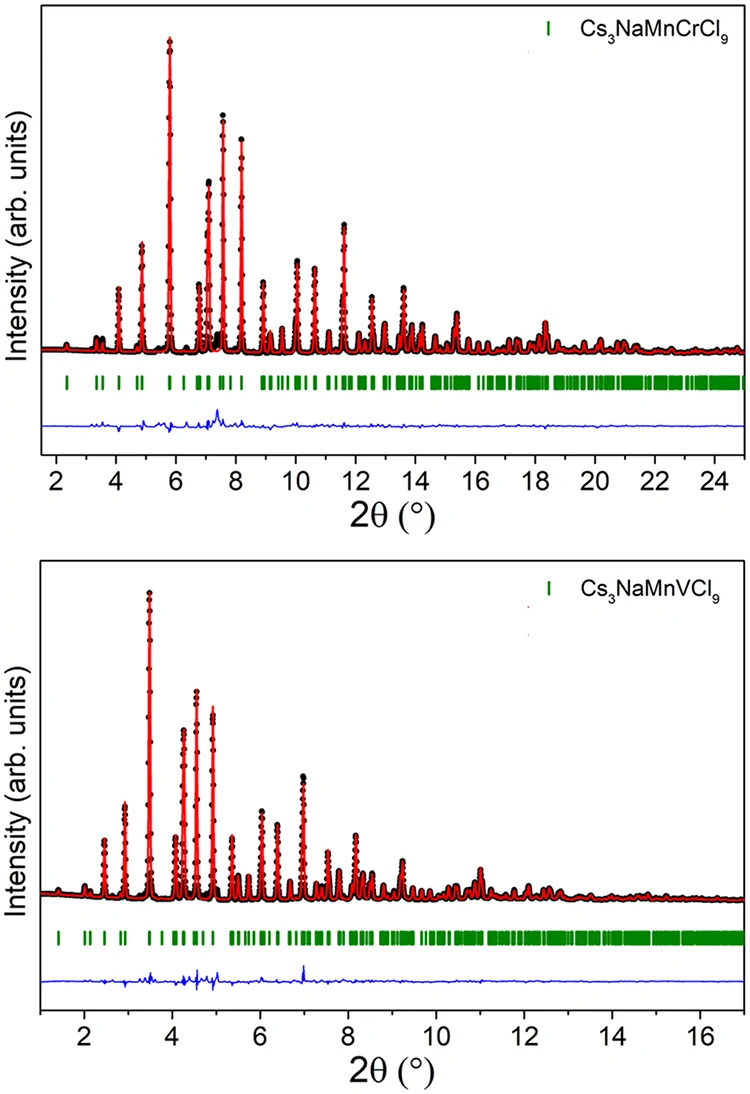
Rietveld fits to synchrotron XRPD data collected on Cs_3_NaMnCrCl_9_ (top) and Cs_3_NaMnVCl_9_ (bottom). The observed data points are denoted by black dots, while the calculated and difference curves are represented as red and blue lines, respectively. Green tick marks indicate the positions of allowed reflections.

**Figure 2:**
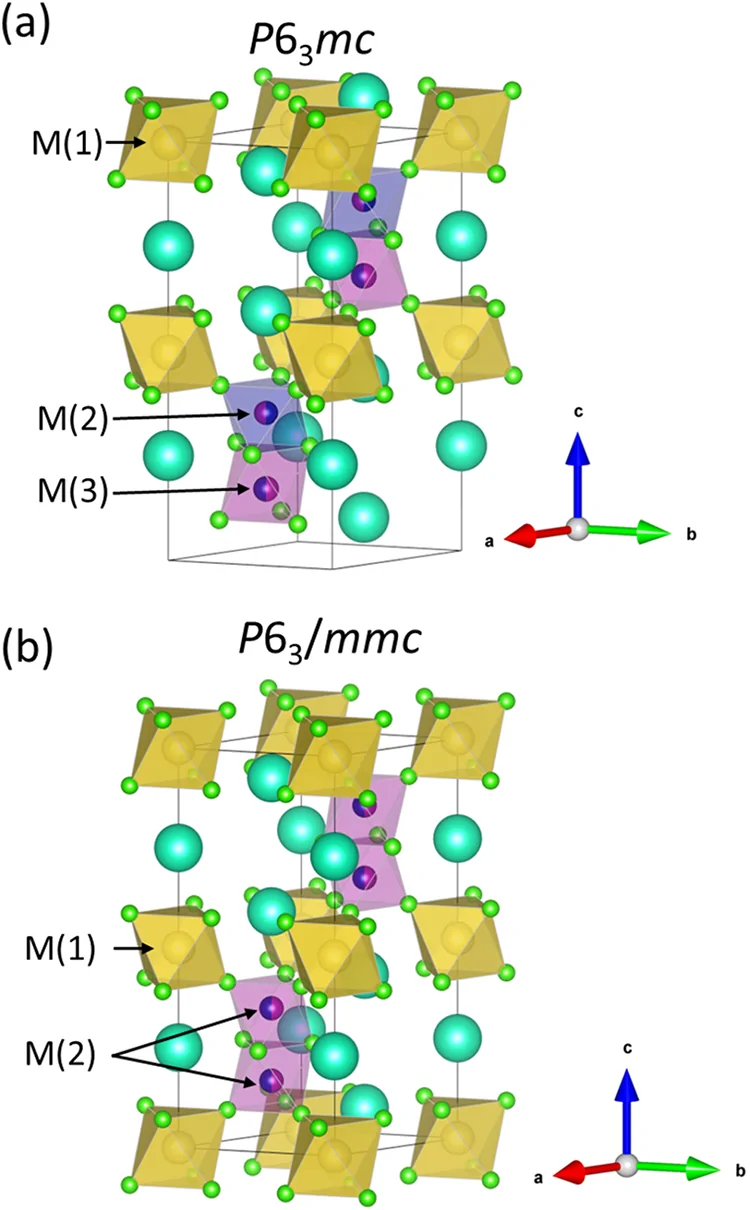
Unit cells of the conventional 6H perovskite structure in the (a) *P*6_3_ *mc* and (b) *P*6_3_/*mmc* models. M(1) refers to the cation in the octahedra that only shares corners, while M(2) and M(3) refer to the cations in the dimer.

A similar fit to the diffraction patterns can be obtained using either the *P*6_3_/*mmc* (centrosymmetric) or the *P*6_3_ *mc* (noncentrosymmetric) space group. The notable difference between the two is the absence of mirror planes perpendicular to the *c*-axis in the latter. This results in two crystallographically unique sites in the M_2_Cl_9_ dimer (Figure 2a) permitting ordering of Mn^2+^ and V^3+^/Cr^3+^, whereas the two cation sites are crystallographically equivalent in *P*6_3_/*mmc* (Figure 2b). If the transition metal ions are randomly distributed, *P*6_3_/*mmc* symmetry is expected, whereas long-range ordering of the Mn^2+^ and V^3+^/Cr^3+^ ions will lower the symmetry to *P*6_3_ *mc*. Unfortunately, the similar X-ray scattering powers of Mn^2+^, Cr^3+^ and V^3+^ render XRPD relatively insensitive to the site ordering, or lack thereof, of these cations.

Starting with Cs_3_NaMnCrCl_9_, a comparative refinement was performed using structural models corresponding to each of the above space groups. A slightly improved fit was obtained with the *P*6_3_ *mc* model (*R*_wp_ = 8.32 vs 8.21%), but a slight improvement in fit is to be expected due to the increased number of variables associated with the lower symmetry space group. To assess the cation distribution in more rigorous manner we turn to neutron diffraction, where Mn^2+^ and Cr^3+^ possess distinctly different coherent scattering lengths (−3.73 fm for Mn vs +3.63 fm for Cr). Interestingly, the positions of the cations that occupy the M(2) site could not be accurately determined from analysis of the NPD data. In fact, the goodness of fit hardly changes if the Mn and Cr sites are left completely vacant. This unusual behavior can be attributed to the opposite signs, but nearly equivalent magnitudes, of the neutron scattering lengths of Mn and Cr. Because of this relationship a 50:50 statistical mixture of the two ions would result in near zero scattering from the M(2) site. Conversely, if the two cations were ordered their scattering lengths should provide sufficient contrast to determine accurate positions and occupancies in the *P*6_3_ *mc* model. When the occupancies are fixed to impose complete site ordering, the quality of the fit degrades significantly. Therefore, we can confidently conclude there is no appreciable long-range order of Mn and Cr ions and hereafter adopt a structural model with *P*6_3_/*mmc* symmetry and a statistical distribution of Mn and Cr on the M(2) site. Further details are given in the Supporting Information.

Neutron diffraction data were not obtained for Cs_3_NaMnVCl_9_, so our analysis of cation order is based solely on synchrotron XRPD data. While there is a slight improvement in the fit obtained with the *P*6_3_ *mc* space group (*R*_wp_ = 3.49 vs 3.39%), similar behavior was seen in our analysis of Cs_3_NaMnCrCl_9_ and in our previous work on Cs_3_NaMnFeCl_9_. Yet, in both cases, NPD analysis and/or single crystal XRD analysis rules out long-range cation order. Additionally, V^3+^ (0.64 Å) has an ionic radius that lies between that of Cr^3+^ (0.615 Å) and Fe^3+^ (0.645 Å),^15^ so it would be surprising if Mn^2+^ and V^3+^ were to adopt an ordered distribution when Mn^2+^ and either Cr^3+^ or Fe^3+^ do not. Therefore, we assume that the transition metal ions in Cs_3_NaMnVCl_9_ do not possess long-range order and the crystal structure can be assigned as having *P*6_3_/*mmc* symmetry, isostructural to Cs_3_NaMnCrCl_9_. A summary of the crystallographic data is given in Table 1. Full details are given in the Supporting Information (Tables S1–S3). It should be noted that even in the absence of long-range cation order, local bonding considerations suggest that bimetallic dimers containing one Mn^2+^ and one M^3+^ cation will be strongly favored.

**Table 1:** Details of the Crystal Structures of Cs_3_NaMnCrCl_9_ and Cs_3_NaMnVCl_9_ ^a^

composition	Cs_3_NaMnCrCl_9_	Cs_3_NaMnVCl_9_
Method	synchrotron XRPD	synchrotron XRPD
λ (Å)	0.3719	0.2241
Space group	*P*6_3_/*mmc*	*P*6_3_/*mmc*
*R*_wp_ (%)	8.322	3.492
*a* (Å)	7.3456(1)	7.36000(8)
*c* (Å)	18.1231(4)	18.1510(4)
volume (Å^3^)	846.87(3)	851.50(3)
density (g cm^–3^)	3.3244(1)	3.3022(1)
Occupancies		
M(1)	1.0 Na	1.0 Na
M(2)	0.50 Mn	0.50 Mn
	0.50 Cr	0.50 V
Bond Distances and Angles		
M(1)–Cl distance	6 × 2.758(4) Å	6 × 2.748(3) Å
M(2)–Cl distances	3 × 2.375(5) Å	3 × 2.386(3) Å
	3 × 2.501(5) Å	3 × 2.549(3) Å
Mn–M(2) distance	3.292(7) Å	3.323(5) Å
M(1)–Cl–M(2) angle	175.8(2)°	176.1(1)°
M(2)–Cl–M(2) angle	82.3(2)°	81.4(1)°

a M­(1) denotes the cation that resides in the octahedron that only shares corners, while M(2) denotes the site in the M_2_Cl_9_ face-sharing bioctahedron.

### Optical Absorption

To evaluate the optical properties, the samples were studied using diffuse reflectance spectroscopy over the range 250–2500 nm (Figure 3). The absorption spectra are dominated by a mix of excitations that involve the octahedrally coordinated transition metal ions. Although the ligand environment of the cations in the dimer is trigonally distorted, which splits the t_2g_ states into an a_1g_ and e_g_ states,^16^ the spectral features can be largely interpreted with a model that assumes a regular octahedral environment.

**Figure 3:**
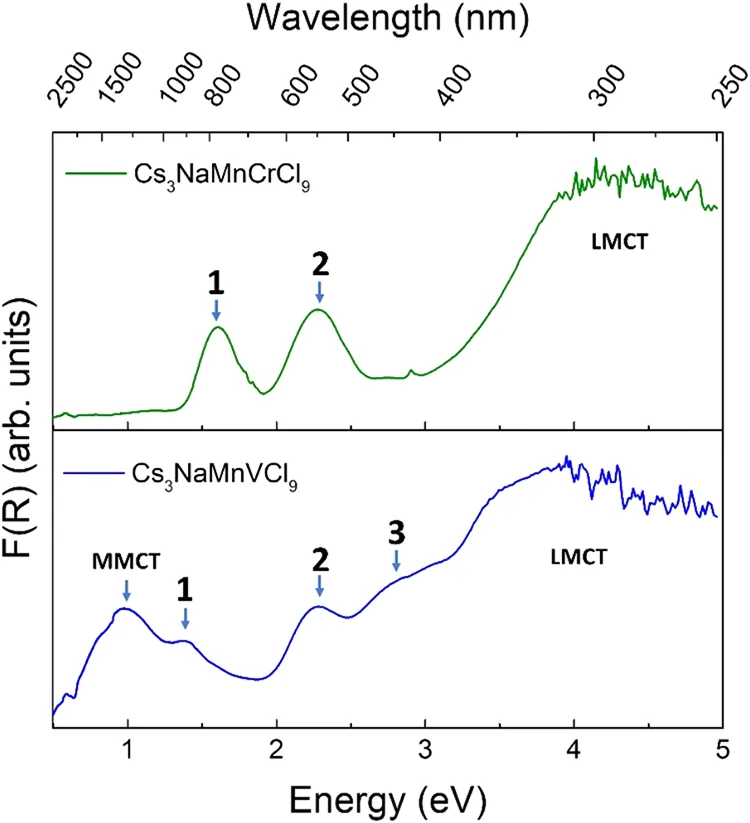
Optical absorption spectra of Cs_3_NaMnCrCl_9_ and Cs_3_NaMnVCl_9_ from 250 to 2500 nm. The d-to-d transitions are labeled as follows: Cs_3_NaMnCrCl_9_ (1) ^4^A_2g_ → ^4^T_2g_(F), (2) ^4^A_2g_ → ^4^T_1g_(F), and for Cs_3_NaMnVCl_9_ (1) ^3^T_1g_ → ^3^T_2g_(F), (2) ^3^T_1g_ → ^3^T_1g_(P), (3) ^3^T_1g_ → ^3^A_2g_(F).

To analyze the spectra in detail, it is instructive to break down the absorption contributed by the metals separately. We start with the Mn^2+^ cation that is present in both compounds. The strongest absorption is expected to be a ligand (Cl) to metal (Mn) charge transfer (LMCT) in the UV region of the spectrum. In the 9R perovskite, CsMnCl_3_, the LMCT transition has an onset of absorption at ∼260 nm (Figure S4). Due to the high-spin d^5^ configuration of Mn^2+^ there are no spin-allowed d-to-d transitions. Thus, at wavelengths longer than the LMCT transition only weak, spin-forbidden d-to-d transitions are observed, leading to the typical pinkish-white color associated with many Mn^2+^ containing compounds. The most prominent spin-forbidden d-to-d transitions are labeled in Figure S4, using assignments from a recent study of CsMnCl_3_ nanocrystals.^17^ In the Cs_3_NaMnCrCl_9_ spectrum some weak features associated with spin-forbidden Mn^2+^ d-to-d transitions can be discerned. The sharp (but very weak) band at ∼420 nm can be assigned to the ^6^A_1_(S) → ^4^A_1_(G) and/or ^6^A_1_(S) → ^4^E­(G) transitions, while the broader feature at slightly lower energy (∼450 nm) can be assigned to the ^6^A_1_(S) → ^4^T_2_(G) transition. In the Cs_3_NaMnVCl_9_ spectrum, other types of electronic transitions are present in the blue region of the spectrum that mask the weak Mn^2+^ d-to-d transitions.

Next, we turn to the d-to-d transitions associated with the [MCl_6_]^3–^ octahedra, beginning with Cs_3_NaMnCrCl_9_. We see two absorption peaks centered at ∼770 and ∼540 nm. These bands are typical of spin-allowed d-to-d transitions in octahedral Cr^3+^ complexes. They can be assigned to the ^4^A_2g_ → ^4^T_2g_(F) and ^4^A_2g_ → ^4^T_1g_(F) transitions, respectively. The positions of these bands are in excellent agreement with d-to-d transitions previously reported for Cr^3+^ ions doped into Cs_2_NaScCl_6_, where absorption peaks are seen at nearly the same positions.^18^ Based on the energies of these two absorption bands, the crystal field splitting (Δ_O_) and Racah parameter (B) are estimated to be 1.61 eV (13,000 cm^–1^) and 0.069 eV (555 cm^–1^), respectively. A third spin-allowed d-to-d band, ^4^A_2g_ → ^4^T_1g_(P), is expected. Using the d^3^ Tanabe-Sugano diagram, we estimate the position of this band to be centered at approximately ∼350 nm (3.6 eV). It is formally a two-electron transition (t_2g_^3^ → t_2g_^1^e_g_^2^) and as such is expected to be considerably weaker than the other two spin-allowed transitions. This fact, coupled with its overlap with the LMCT transition, makes it impossible to resolve from reflectance data. The lack of significant absorption in the blue and red regions of the spectrum explains the purple color of this compound.

Turning our attention to the Cs_3_NaMnVCl_9_ spectrum, three spin-allowed d-to-d transitions are expected for a d^2^ cation in an octahedral field. To assign these bands, we refer to prior studies of chloride double perovskites containing V^3+^. When V^3+^ is doped into Cs_2_NaYCl_6_ spin-allowed d-to-d transitions from the ^3^T_1g_ ground state are observed at 900, 520, and 425 nm.^19^ More recent studies of the cubic double perovskite Cs_2_NaVCl_6_ reported d-to-d transitions at ∼900 nm and ∼560 nm, and a ruby red coloration similar to what we observe for Cs_3_NaMnVCl_9_.^20^ By comparison we assign the peaks centered at 940 and 560 nm to the ^3^T_1g_ → ^3^T_2g_(F) and ^3^T_1g_ → ^3^T_1g_(P) transitions of an octahedrally coordinated V^3+^ ion, respectively. Using these values with the d^2^ Tanabe-Sugano diagram, the octahedral ligand-field splitting Δ_O_ and Racah parameter B are estimated to be 1.44 eV (11,600 cm^–1^) and 0.068 eV (550 cm^–1^), respectively. The fact that Δ_O_ is smaller than it is for Cr^3+^ is consistent with the slightly longer M(2)–Cl bond distances found in Cs_2_NaMnVCl_9_ (Table 1). The values of B found here are consistent with prior studies of octahedrally coordinated Cr^3+^ and V^3+^ ions surrounded by chloride ions, where values of 600 ± 50 cm^–1^ are typical.^18,23^ From the Tanabe-Sugano parameters we can estimate the wavelength of the ^3^T_1g_ → ^3^A_2g_(F) transition to be ∼450 nm, where a weak peak is seen when fitting the spectrum (Figure S7). This transition is considerably weaker than the other spin-allowed d-to-d transitions because it involves the simultaneous excitation of two electrons into the e_g_ orbitals (i.e., t_2g_^2^e_g_^0^ → t_2g_^0^e_g_^2^). A summary of the d-to-d transitions in both compounds is provided in Table 2. It is interesting to note that despite the fact that the bond distances given in Table 1 suggest a reasonably large trigonal distortion of the octahedra, splitting of the d-to-d transitions is not readily apparent in the reflectance spectra. One possible explanation is that the local structure may consist of a fairly symmetric M^3+^ octahedron and a strongly distorted Mn^2+^ octahedron. This is seen in the structures of Cs_3_NaMnRhCl_9_ and Cs_3_NaFeRhCl_9_ where the M^2+^ and M^3+^ exhibit long-range ordering.^7^

**Table 2:** d-to-d Transitions Determined from Fitting Diffuse Reflectance Spectra with Gaussian Peaks as well as the Δ_O_ and *B* Values Corresponding to the M^3+^Cl_6_ Octahedron^a^

	M^3+^ = Cr	M^3+^ = V
Peak 1		
assignment	^4^A_2g_ → ^4^T_2g_(F)	^3^T_1g_ → ^3^T_2g_(F)
wavelength (nm)	770	940
Peak 2		
assignment	^4^A_2g_ → ^4^T_1g_(F)	^3^T_1g_ → ^3^T_1g_(P)
wavelength (nm)	540	560
Peak 3		
assignment	^4^A_2g_ → ^4^T_1g_(P)	^3^T_1g_ → ^3^A_2g_(F)
wavelength (nm)	∼350	∼450
Δ_O_	1.61 eV 13,000 cm^–1^	1.44 eV 11,600 cm^–1^
*B*	0.069 eV 555 cm^–1^	0.068 eV 550 cm^–1^

a In both compounds, the position of the highest energy transition (peak 3) was estimated using the corresponding Tanabe-Sugano diagram.

The LMCT transitions in these compounds are found in the near UV and high energy visible regions of the spectrum. A qualitative estimate of the LMCT transitions can be made from Figure 4, which shows an overlay of the spectra from Cs_3_NaMnM^3+^Cl_9_ (M^3+^ = V, Cr), the isostructural Cs_3_NaMnFeCl_9_,^6^ and CsMnCl_3_. Proceeding from the UV to longer wavelengths we can estimate the onset energy of the LMCT transition by extrapolating the sharp drop in absorbance to the background. These energies can also be considered equivalent to the bandgap. Doing so allows us to qualitatively order the relative energies of the LMCT transitions as CsMnCl_3_ (260(10) nm, 4.8(2) eV) > Cs_3_NaMnCrCl_9_ (399(6) nm, 3.11(5) eV) > Cs_3_NaMnVCl_9_ (462(7) nm, 2.68(4) eV) > Cs_3_NaMnFeCl_9_ (620(6) nm, 2.00(2) eV). These values are in reasonably good agreement with the published bandgaps of hexagonal and cubic halide perovskites containing the same transition metal ions: CsMnCl_3_ *E*_g_ = 4.6 eV, Cs_2_NaVCl_6_ *E*_g_ = 2.6 eV, and Cs_2_NaFeCl_6_ *E*_g_ = 2.2 eV.^6,10,20,21^ The lower energy LMCT transition in Cs_3_NaMnVCl_9_ results in significant absorption in the violet and blue regions of the spectrum and is partially responsible for differences in color between the purple Cs_3_NaMnCrCl_9_ and the red Cs_3_NaMnVCl_9_.

**Figure 4:**
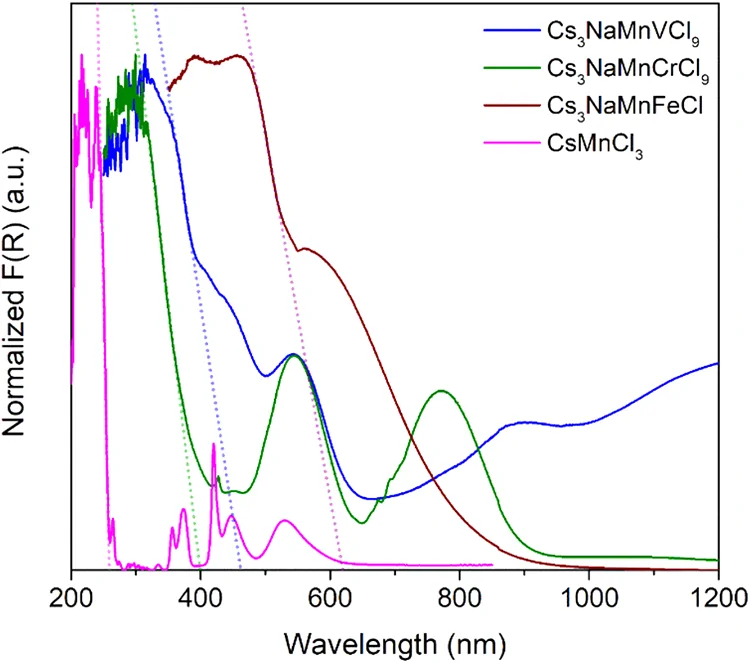
Optical absorption spectra of Cs_3_NaMnM^3+^Cl_9_ (M^3+^ = V, Cr, Fe) and CsMnCl_3_ from 200–1000 nm. The dashed lines represent extrapolation of the LMCT transitions to the baseline.

Two absorption features remain unaccounted for in the Cs_3_NaMnVCl_9_ spectrum: a broad peak in the infrared centered at ∼1390 nm, and a low energy shoulder on the LMCT transition at ∼420 nm. Since the d-to-d transitions are accounted for, it is likely that these bands are either of MMCT or LMCT character. It is instructive to compare the absorption spectrum of Cs_3_NaMnVCl_9_ with related chloride perovskites containing V^3+^ where MMCT transitions can be ruled out, such as Cs_2_NaVCl_6_ (cubic double perovskite), Cs_2_LiVCl_6_ and Rb_2_LiVCl_6_ (2H hexagonal perovskites).^22^ These compounds do not show any absorption peaks at wavelengths longer than the ^3^T_1g_ → ^3^T_2g_(F) transition at 900–950 nm. From this observation we assign the broad peak at ∼1390 nm as a Mn^2+^ → V^3+^ MMCT band with an energy on the order of 0.95 eV. While this assignment is based on indirect evidence, both d-to-d and LMCT transitions can be ruled out, leaving an MMCT transition as the only viable assignment. The other unassigned absorption peak falls at ∼420 nm, on the shoulder of the LMCT transition. It seems unlikely that there would be two MMCT transitions separated by such a large energy. It is more likely that the t_2g_^2^e_g_^0^ electron configuration of V^3+^ leads to multiple LMCT transitions and thus a more complicated shape in the blue region of the spectrum.

### Magnetism

When discussing the magnetism of 6H perovskites, it is helpful to start by considering the most prominent short-range magnetic interactions. The nearest neighbor interaction (*J*_1_) is the short intradimer magnetic exchange between M^2+^ and M^3+^ (Figure 5a). Two different exchange pathways contribute to *J*_1_: M^2+^–Cl–M^3+^ superexchange coupling and the competing direct-exchange pathway. The 90° superexchange between a metal with half-filled e_g_ orbitals (Mn^2+^) and another with empty e_g_ orbitals (Cr^3+^, V^3+^) is expected to be antiferromagnetic (AFM) in nature. The competing direct exchange is driven by the overlap of the t_2g_ orbitals and is also AFM. Hence, the two exchange mechanisms are expected to work cooperatively to give AFM nearest neighbor *J*_1_ coupling.

**Figure 5:**
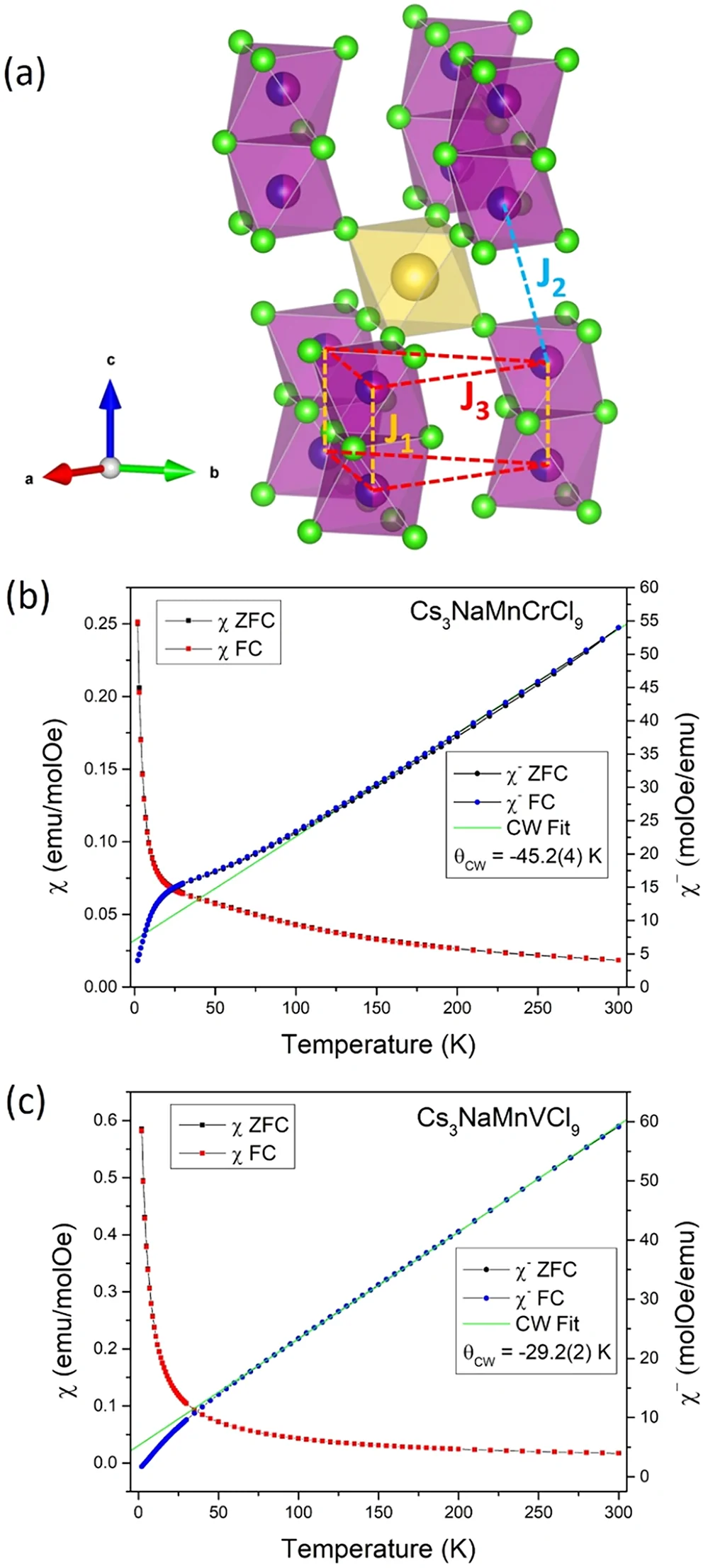
(a) Depiction of the *J*_1_, *J*_2_, and *J*_3_ magnetic pathways expected in Cs_3_NaMnVCl_9_ and Cs_3_NaMnCrCl_9_. Magnetic susceptibility and inverse susceptibility of (b) Cs_3_NaMnVCl_9_ and (c) Cs_3_NaMnCrCl_9_ collected from 2 K–300 K.

The second (*J*_2_) and third-nearest neighbor (*J*_3_) exchanges are interdimer interactions that are typically weak due to the longer superexchange pathways. To visualize these pathways, it is useful to describe the structure as consisting of layers of bioctahedral dimers arranged in the *ab*-plane in pattern commonly referred to as a triangular lattice. The *J*_2_ interaction is between M_2_Cl_9_ dimers in neighboring layers and the *J*_3_ interaction is between dimers within the same layer. Their magnitudes are expected to be much smaller than the intradimer *J*_1_ interaction.^7,8^

DC magnetic susceptibility measurements from 2–300 K are shown in Figure 5b,c. Starting with Cs_3_NaMnCrCl_9_, the magnetic susceptibility curve shows no sign of long-range magnetic order down to 2 K. The high temperature region (150–300 K) of the inverse susceptibility is well fit to the Curie–Weiss (CW) law and yields a Weiss temperature (θ_CW_) of −45.2 K. This indicates dominant AFM interactions, which can be expected from the AFM nature of the *J*_1_ interaction. From the fit, we obtain an effective moment (μ_eff_) of 7.17 μ_B_ per formula unit, not far from the 7.07 μ_B_ expected for a spin-only system containing *S* = 3/2 and 5/2 ions. At temperatures lower than 150 K, a negative deviation from the CW law is observed until ∼10 K where the susceptibility begins to increase sharply. The sharp increase is characteristic of a “Curie tail,” which is typically associated with a magnetic impurity and may be related to the unknown secondary phase(s) identified in the diffraction patterns. The deviation from the CW law may suggest that the compound is approaching long-range antiferromagnetic order. In some compounds with 6H type structures, such as Cs_3_Fe_2_X_9_, a decreased susceptibility relative to the CW law is observed when approaching the Néel temperature.^23,24^

The magnetic susceptibility of Cs_3_NaMnVCl_9_ also gives no indication of long-range magnetic order over the temperature range investigated. The inverse susceptibility can be fit to the CW law over the temperature window 50–300 K. This fitting gives a Weiss constant, θ_CW_ of −29.2 K. This value is smaller (in absolute value) than that of the Cr-analogue which suggests weaker overall AFM interactions. The effective moment, μ_eff_ = 6.66 μ_B_ per formula unit, is in good agreement with the spin-only expected moment of 6.56 μ_B_ for a system containing *S* = 1 and 5/2 ions. At temperatures lower than ∼50 K a positive deviation from the CW law is observed in the magnetic susceptibility, which interestingly is opposite of the deviation found in Cs_3_NaMnCrCl_9_.

## Discussion

It is well-known that the interaction between cations within the M_2_X_9_ face-sharing bioctahedron is a balance between electrostatic repulsions and attractions that can arise from metal–metal bonding. An analysis of the bond distances and angles within the bioctahedral cluster is instructive in judging the net interaction. Here we find both cations are displaced away from the shared face, as expected if electrostatic repulsions are dominant. This is apparent from the shorter bond distances to chloride ions in the unshared face of the dimer, 2.375(5) Å (Cr) and 2.386(3) Å (V), than to chloride ions in the shared face, 2.501(5) Å (Cr) and 2.549(3) Å (V). Further insight can be gained by the analyzing the M(2)–Cl–M(2) angle. The bond angle in both compounds is considerably more obtuse than the ideal angle of 70.53° expected for undistorted octahedra sharing a common face. An angle smaller than 70.53° is characteristic of metal–metal bonding that is facilitated by the overlap of partially filled t_2g_ orbitals. Although such an acute angle is unusual, it has been observed in the vacancy-ordered 6H perovskites Cs_3_Mo_2_Cl_9_ and Cs_3_W_2_Cl_9_, where metal–metal bonding is appreciable.^25,26^ A bond angle much larger than 70.53° is indicative of a repulsive interaction between cations, for example in Cr_2_O_3_ it is 82.74°.^27^ The M(2)–Cl–M(2) angles seen here, both >81°, are further evidence for repulsive interactions between cations. Finally, and perhaps most importantly, the absence of any metal–metal bonding is consistent with Mn^2+^–M^3+^ distances of ∼3.3 Å, which are much longer than the interatomic distances seen in elemental Mn (2.2–2.9 Å), Cr (∼2.5 Å), or V (∼2.6 Å).^28–30^ The metal–metal distances seen here are also significantly larger than in comparable oxides containing Cr^3+^ ions in the bioctahedra, such as Ba_3_Cr_2_MoO_9_ where the Cr–Cr distance is 2.52 Å.^31^ The lack of metal–metal bonding can be attributed to a combination of the limited radial extension of the 3d orbitals and the relatively large radius of the chloride ion that separates the metals.

The absence of strong intradimer magnetic coupling is also evident in the magnetic properties of compounds in the Cs_3_NaMnM^3+^Cl_9_ (M^3+^ = V, Cr, Fe) series. The temperature dependent magnetic susceptibility can be well modeled with a Curie–Weiss fit, consistent with localized moments that change minimally with temperature. This is unlike the vacancy-ordered 6H perovskites Cs_3_M_2_Cl_9_ (M = Cr^3+^, Mo^3+^, W^3+^) or oxides such as Ba_3_Cr_2_MO_9_ (M = Mo^6+^, W^6+^),^25,26,31,32^ where the presence of strong antiferromagnetic coupling within the bioctahedron lead to the formation of spin dimers and effective moments that are strongly temperature dependent. In the Cs_3_NaMnMCl_9_ series, the effective moments obtained from the Curie–Weiss fits are very close to the expected spin-only values. This not only confirms the validity of treating the system as weakly coupled, temperature-independent individual ion spins, it reveals the lack of appreciable spin–orbit coupling. The parameters obtained from the CW fits are given in Table 3.

**Table 3:** Values Corresponding to CW Fit of the Magnetic Susceptibility of Cs_3_NaMnVCl_9_, Cs_3_NaMnCrCl_9_

compound	Cs_3_NaMnVCl_9_	Cs_3_NaMnCrCl_9_	Cs_3_NaMnFeCl_9_
CW fit range	50–300 K	150–300 K	50–300 K
θ_CW_ (K)	–29.2(2)	–45.2(4)	–41.4(1)
*C* (cm^3^·K·mol^–1^)	5.539(5)	6.41(1)	8.665(5)
μ_eff_ (μB)	6.657(3)	7.168(6)	8.326(2)
Expected μ_eff_ (spin only)	6.56	7.07	8.367
ref	This work	This work	^6^

The presence of chemical disorder and the lack of large single crystals suitable for magnetic measurements make it impractical to extract values for the individual exchange interactions depicted in Figure 5a, but the Weiss constants give important clues about the sign and strength of the exchange interactions in these materials. The magnetism of Cs_3_NaMnFeCl_9_ is perhaps the easiest to interpret because both Mn^2+^ and Fe^3+^ have the same 3d^5^ electron configuration, so the lack of long-range cation order should have no impact on the sign of the exchange interactions. The *J*_2_ and *J*_3_ pathways Mn^2+^–Cl–Na–Cl–Fe^3+^ will result in antiferromagnetic (AFM) superexchange coupling. A conclusion that is robust against Mn^2+^/Fe^3+^ disorder. The intradimer exchange, *J*_1_, is a competition between ferromagnetic (FM) ∼90° superexchange through the chloride ligand and AFM direct exchange across the shared face. In the vacancy-ordered 6H perovskite Cs_3_Fe_2_Cl_9_, where Fe^3+^ occupies both positions within the Fe_2_Cl_9_^3–^ dimer, the intradimer exchange is thought to be FM, signaling that Fe–Cl–Fe superexchange is stronger than Fe–Fe direct exchange, while the longer range *J*_2_ interaction is unambiguously AFM. Although *J*_1_ (0.254 meV) is estimated to be more than twice *J*_2_ (0.116 meV), the number of *J*_2_ pathways outnumbers *J*_1_ by a 6:1 ratio, so the Weiss constant is negative, θ_CW_ ≈ −15 K. The magnetic interactions in Cs_3_NaMnFeCl_9_ should be similar, but the replacement of half of the Fe^3+^ ions with Mn^2+^ ions should weaken the superexchange coupling, due to the reduction in covalency of the metal-chloride bond. The sign of the *J*_2_ and *J*_3_ interactions should be the same in both compounds. The strength of these long-range superexchange interactions will be weakened by the presence of Mn^2+^. If we assume similar *J*_2_ and *J*_3_ interactions in both compounds, the observation of a Weiss constant in Cs_3_NaMnFeCl_9_ that is significantly more negative, θ_CW_ = −41 K, is a clear indication that the intradimer exchange, *J*_1_, is AFM. From that we can conclude that the AFM direct exchange is stronger than the competing FM superexchange.

The Weiss constant of −45 K observed for Cs_3_NaMnCrCl_9_ is similar to that of Cs_3_NaMnFeCl_9_. However, the similarity may be something of a coincidence. If we were to assume a locally ordered arrangement of MnCrCl_9_^4–^ bioctahedra, the Mn^2+^–Cl–Na–Cl–Cr^3+^ interdimer exchange (*J*_2_ and *J*_3_) should be FM, given the d^5^ and d^3^ configurations of Mn^2+^ and Cr^3+^. However, if the orientation of a MnCrCl_9_^4–^ bioctahedron is reversed, one would expect AFM interdimer exchange, via Mn^2+^–Cl–Na–Cl–Mn^2+^ and Cr^3+^–Cl–Na–Cl–Cr^3+^ exchange pathways. The lack of long-range cation order will produce a mixture of the FM and AFM interactions. Summing over all interdimer exchange pathways should lead to a near cancellation. If that assumption is valid, the Weiss constant is derived largely from *J*_1_ and the AFM intradimer exchange must be stronger in Cs_3_NaMnCrCl_9_ than it is in Cs_3_NaMnFeCl_9_. This is expected given that within a bioctahedral dimer the 90° Mn^2+^–Cl–Cr^3+^ superexchange and the Mn^2+^–Cr^3+^ direct exchange are both AFM and act cooperatively. Moving further right in the periodic table to Cs_3_NaMnVCl_9_, the reduced Weiss constant (θ_CW_ = −29 K) can be partially explained by the reduction in the local moment when Cr^3+^ (*S* = 3/2) is replaced by V^3+^ (*S* = 1), and partially due to a reduction in strength of the *J*_1_ exchange interaction.

Next, we turn our attention to the optical properties. Both compounds feature d-to-d transitions that can be adequately described by assuming an octahedral crystal field and using Tanabe-Sugano diagrams. The bond distances given in Table 1 reveal reasonably large trigonal distortions of the octahedra, so some splitting of the bands is expected. However, in Cs_3_NaMnCrCl_9_ two of the three d-to-d transitions are well resolved from other optical transitions and the splitting of those peaks is not resolved. From this we can infer that splitting of the d-to-d transitions is also likely to be small in Cs_3_NaMnVCl_9_.

The LMCT transition gives a measure of the relative energies of the 3d orbitals of the transition metal ions with respect to the 3p orbitals of the surrounding chloride ions. Recounting the onset energies of the LMCT transitions from the results section: Cl^–^ → Mn^2+^ (CsMnCl_3_, 4.8 eV) > Cl^–^ → Cr^3+^ (Cs_3_NaMnCrCl_9_, 3.1 eV) > Cl^–^ → V^3+^ (Cs_3_NaMnVCl_9_, 2.7 eV) > Cl^–^ → Fe^3+^ (Cs_3_NaMnFeCl_9_, 2.0 eV). The trends in LMCT energy can largely be rationalized based on the effective electronegativity of the transition metal ions in question. The 3d orbitals of the +3 ions experience a greater effective nuclear charge and therefore exhibit a higher effect electronegativity than Mn^2+^, leading to a lower energy LMCT transition. We can use Jorgensen’s concept of optical electronegativity to qualitatively predict the energetic order of the Cl → M^3+^ LMCT transitions.^33,34^ The formulas for estimating the energy for a transition from the ligand 3p π orbitals into the metal t_2g_ orbitals can be written in terms of optical electronegativity of the chloride ligand, χ­(Cl), and the transition metal, χ­(M), as well as the spin-pairing energy parameter *D*, which can be approximated as ≈7*B*. The inclusion of the latter term is necessary to account for changes in the interelectronic repulsion energy within the d shell and between the d shell and the ligand hole that is created by the LMCT transition. The equations for estimating the LMCT energy vary by the electron count of the transition metal ion and are as follows

$$\begin{array}{l} \mathrm{LMCT}\;\mathrm{Energy}\;\left(\mathrm{in}\;{\mathrm{cm}}^{-1}\right)=30,000\left[\chi \left(\mathrm{Cl}\right)-\chi \left(M\right)\right]+\left(8/3\right)D,\mathrm{for}\;a\;d^{5}\;\mathrm{ion}\;\left(i.e.\;{\mathrm{Fe}}^{3+}\right)\\ \mathrm{LMCT}\;\mathrm{Energy}\;\left(\mathrm{in}\;{\mathrm{cm}}^{-1}\right)=30,000\left[\chi \left(\mathrm{Cl}\right)-\chi \left(M\right)\right]+\left(2\right)D,\mathrm{for}\;a\;d^{3}\;\mathrm{ion}\;\left(i.e.\;{Cr}^{3+}\right)\\ \mathrm{LMCT}\;\mathrm{Energy}\;\left(\mathrm{in}\;{\mathrm{cm}}^{-1}\right)=30,000\left[\chi \left(\mathrm{Cl}\right)-\chi \left(M\right)\right]-\left(4/3\right)D,\mathrm{for}\;a\;d^{2}\;\mathrm{ion}\;\left(i.e.\;V^{3+}\right) \end{array}$$

The optical electronegativities are scaled to correspond with the Pauling electronegativity scale and are 3.0 for Cl^–^, 2.5 for Fe^3+^, 1.9 for Cr^3+^, and 1.9 for V^3+^.^34^ To make crude estimates of the LMCT energy we take *B* = 600 cm^–1^ and *D* = 4200 cm^–1^ for all three ions. Doing so gives LMCT energies of 26,200 cm^–1^ (3.2 eV) for Cl^–^ → Fe^3+^, 41,400 cm^–1^ (5.1 eV) for Cl^–^ → Cr^3+^, and 27,400 cm^–1^ (3.4 eV) for Cl^–^ → V^3+^. When comparing these estimates with our experimental values, bear in mind that these values are for an isolated molecule or complex ion and they represent the peak of the LMCT transition, not the onset of the transition. Even though the quantitative agreement is not terribly good, it does capture the relative order. It does so because exciting an electron from a doubly occupied chloride 3p orbital into the empty V 3d t_2g_ orbital reduces the spin pairing energy and therefore takes less energy than a comparable Cl^–^ → Cr^3+^ transition, even though Cr^3+^ and V^3+^ have similar optical electronegativities. Thus, we can understand why the bandgap of Cs_3_NaMnVCl_9_ is smaller than that of Cs_3_NaMnCrCl_9_.

The energy of the Mn^2+^ → V^3+^ MMCT transition (0.95 eV) is much smaller than the Mn^2+^ → Fe^3+^ MMCT transition (2.2 eV) previously reported in Cs_3_NaMnFeCl_9_.^6^ To understand this red shift we need to consider differences in the orbital energies of V^3+^ and Fe^3+^ as well as changes in Hund’s coupling. Focusing on the t_2g_ orbitals and assuming antiferromagnetic alignment within a bioctahedral dimer, the MMCT transition in both compounds is expected to involve transfer of an up-spin electron on Mn^2+^ into the up-spin manifold on either V^3+^ or Fe^3+^, as illustrated in Figure 6. Based on the optical electronegativites discussed above, Fe^3+^ is more electronegative than V^3+^ and the down-spin Fe t_2g_ orbitals will be lower in energy than the comparable V t_2g_ orbitals. In the absence of other effects, the MMCT in Cs_3_NaMnVCl_9_ would be blue-shifted with respect to Cs_3_NaMnFeCl_9_, possibly into the UV region of the spectrum. Experimentally the opposite is seen, the MMCT is red-shifted into the near-IR. This can be explained by differences in the intra-atomic exchange interactions (Hund’s coupling). Transferring an electron onto Fe^3+^ will result in double occupation of one of the t_2g_ orbitals, whereas the electron that is transferred onto V^3+^ can occupy the empty t_2g_ orbital and therefore experience a much smaller electron–electron repulsion. This is reflected in the reduced splitting of the t_2g_↓ and t_2g_↑ orbitals in Figure 6.

**Figure 6:**
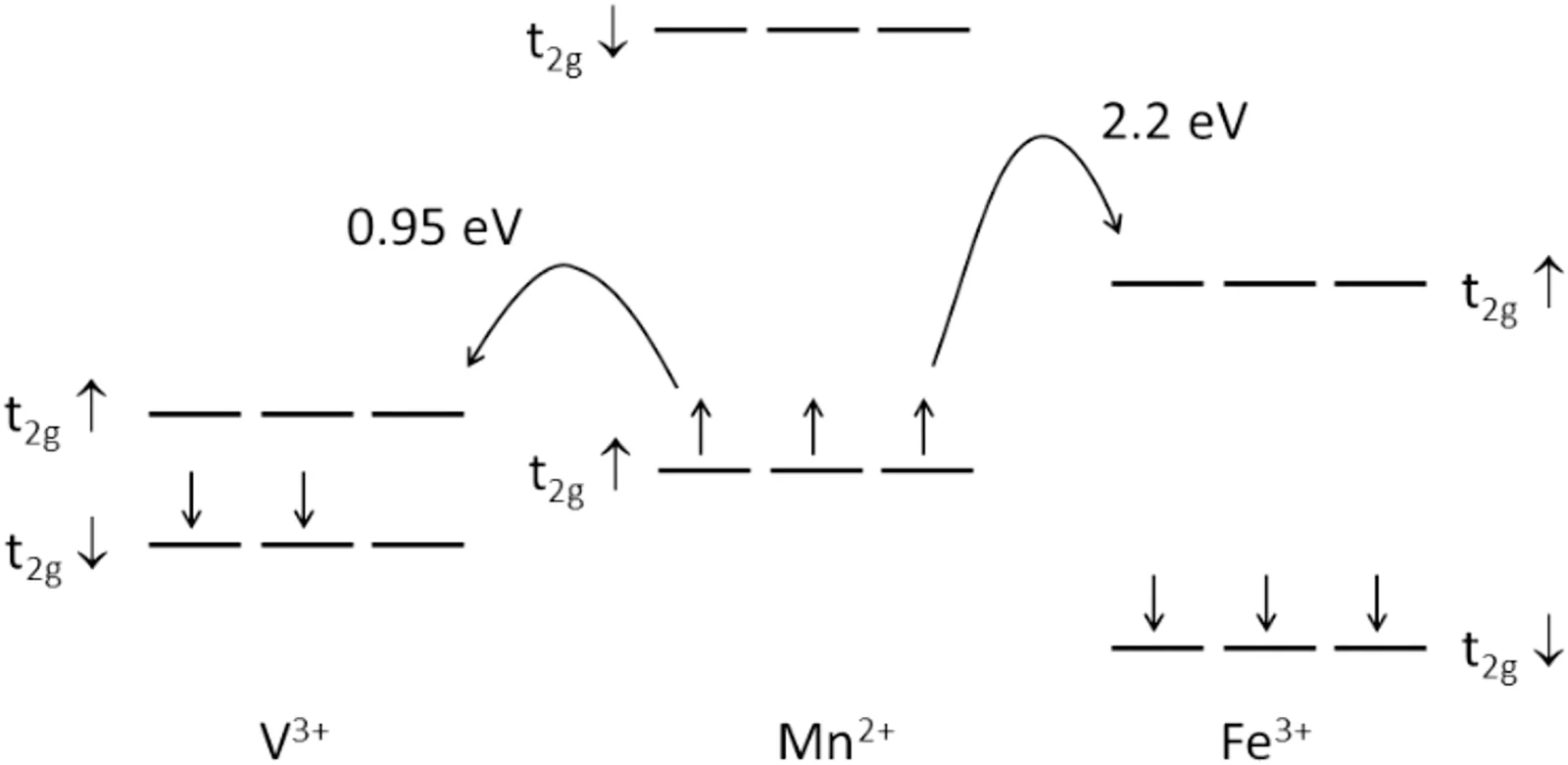
Schematic of the Mn^2+^→M^3+^ MMCT transition in Cs_3_NaMnVCl_9_ (left) and Cs_3_NaMnFeCl_9_ (right).

The absence of a MMCT in Cs_3_NaMnCrCl_9_ is at first glance surprising. Naively one would expect to see such a transition at an energy intermediate between the V^3+^ and Fe^3+^ containing compounds. However, both Cr^3+^ and Fe^3+^ have a half-filled t_2g_ orbital manifold so the changes in intra-atomic exchange that accompany an MMCT transition might reasonably be expected to be similar for Cs_3_NaMnCrCl_9_ and Cs_3_NaMnFeCl_9_. In that case the differences in MMCT energy will come down largely to differences in optical electronegativity. Since Cr^3+^ is more electropositive than Fe^3+^, the MMCT energy in Cs_3_NaMnCrCl_9_ will be larger than in Cs_3_NaMnFeCl_9_. Since there is no reason to expect the MMCT to be absent in Cs_3_NaMnCrCl_9_, the only plausible alternative is that the MMCT peak is blue-shifted into the UV where it overlaps with the LMCT.

## Conclusion

Powders of the 6H perovskites Cs_3_NaMnCrCl_9_ and Cs_3_NaMnVCl_9_ have been characterized in terms of structural, optical, and magnetic properties. Diffraction studies reveal that the Na^+^ ions occupy the corner sharing octahedral positions, while Mn^2+^ and M^3+^ are disordered over the octahedral sites in the face-sharing bioctahedral dimers. The optical absorption for both compounds was studied using diffuse reflectance spectroscopy. Their absorption spectra largely consist of ligand-to-metal charge transfers and spin-allowed d-to-d transitions. Cs_3_NaMnVCl_9_ has an LMCT transition that is smaller than the LMCT for Cs_3_NaMnCrCl_9_ and therefore red-shifted into the visible. It also exhibits a Mn^2+^ → V^3+^ MMCT transition in the IR at ∼1390 nm, whereas the Mn^2+^ → Cr^3+^ MMCT transition in Cs_3_NaMnCrCl_9_ falls in the UV and overlaps with LMCT transitions. SQUID magnetometry shows conventional magnetic behavior with temperature independent, localized moments consistent with expectations for the transition metal ions involved. In both compounds intradimer antiferromagnetic interactions dominate with the Cr-analogue being the stronger of the two due to the higher covalency of the Cr–Cl bonding. Neither sample undergoes long-range magnetic order down to 2 K.

## Supplementary Material



## Notes

Additional details of refinement method and analysis behind space group assignment; parameters obtained from Rietveld refinements on synchrotron XRPD patterns of Cs_3_NaMnCrCl_9_ and Cs_3_NaMnVCl_9_ (Tables S1– S3); Rietveld refinement on a synchrotron XRPD pattern of Cs_3_NaMnCrCl_9_ with arrows identifying reflections belonging to unknown phase(s) (Figure S1); Rietveld refinement on a NPD pattern of Cs_3_NaMnCrCl_9_ (Figure S2); Rietveld refinement on a synchrotron XRPD pattern of Cs_3_NaMnCrCl_9_ with arrows identifying reflections belonging to unknown phase(s) (Figure S3); pseudo-absorbance plot of CsMnCl_3_ with assigned d-to-d transitions (Figure S4); pseudoabsorbance plot of CsMnCl_3_, Cs_3_NaMnCrCl_9_, and Cs_3_NaMnVCl_9_ (Figure S5); fitting of UV–vis-NIR spectra of both compounds (Figures S6 and S7 and Tables S4 and S5) (PDF)

## References

[1] Townsend M. G. (1968). Visible
Charge Transfer Band in Blue Sapphire. Solid
State Commun..

[2] Ferguson J., Fielding P. E. (1971). The Origins of the Colours of Yellow, Green and Blue
Sapphires. Chem. Phys. Lett..

[3] Fuchs Y., Lagache M., Linares J., Maury R., Varret F. (1995). Mössbauer
and Optical Spectrometry of Selected Schörl-Dravite Tourmalines. Hyperfine Interact..

[4] Rossman G. R., Mattson S. M. (1968). Yellow, Mn-rich Elbaite with Mn-Ti Intervalence Charge
Transfer. Am. Mineral..

[5] Smith G. (1977). Low-temperature
Optical Studies of Metal-Metal Charge-Transfer Transitions in Various
Minerals. Can. Mineral..

[6] Liu D., Milder A., Stevens J., Foster C., Beck B., Avdeev M., Woodward P. M. (2025). Temperature-Dependent Mixed Valency
in the Hexagonal Perovskite Cs_3_NaFe_2_Cl_9_. J. Am. Chem. Soc..

[7] Liu D., Foster C. J., Beck B., Woodward P. M. (2025). Triply Ordered 6H
Halide Perovskites: A Family of Triangular Lattice Antiferromagnets. Inorg. Chem..

[8] Xue J., Song R., Guo Z., Sung H. H., Lortz R., Williams I. D., Lu H. (2024). Extended Honeycomb Metal Chloride
with Tunable Antiferromagnetic Correlations. Chem. Mater..

[9] Tsuboi T., Chiba M., Ajiro Y. (1985). Optical Absorption Spectra of the
One-Dimensional Ferromagnet CsFeCl_3_. Phys. Rev. B.

[10] Ji F., Klarbring J., Zhang B., Wang F., Wang L., Miao X., Ning W., Zhang M., Cai X., Bakhit B., Magnuson M., Ren X., Sun L., Fahlman M., Buyanova I. A., Chen W. M., Simak S. I., Abrikosov I. A., Gao F. (2024). Remarkable Thermochromism in the
Double Perovskite Cs_2_NaFeCl_6_. Adv. Opt. Mater..

[11] Coelho A. A. (2018). Topas and
Topas-Academic: An Optimization Program Integrating Computer Algebra
and Crystallographic Objects Written in C++. J. Appl. Crystallogr..

[12] Momma K., Izumi F. (2011). Vesta 3 for Three-Dimensional Visualization
of Crystal, Volumetric
and Morphology Data. J. Appl. Crystallogr..

[13] Kubelka P., Munk F. (1931). A Contribution to the
Optics of Pigments. Z.
Technol. Phys..

[14] Bain G. A., Berry J. F. (2008). Diamagnetic Corrections and Pascal’s Constants. J. Chem. Educ..

[15] Shannon R. D. (1976). Revised
Effective Ionic Radii and Systematic Studies of Interatomic Distances
in Halides and Chalcogenides. Acta Crystallogr.,
Sect. A.

[16] Kugel K. I., Khomskii D. I., Sboychakov A. O., Streltsov S. V. (2015). Spin-Orbital
Interaction for Face-Sharing Octahedra: Realization of a Highly Symmetric
SU(4) Model. Phys. Rev. B.

[17] Matuhina A., Grandhi G. K., Pan F., Liu M., Ali-Löytty H., Ayedh H. M., Tukiainen A., Smått J.-H., Vähänissi V., Savin H., Li J., Rinke P., Vivo P. (2023). Role of CsMnCl_3_ Nanocrystal
Structure on Its Luminescence Properties. ACS
Appl. Nano Mater..

[18] Wenger O. S., Güdel H. U. (2001). Optical Spectroscopy of CrCl_6_
^3–^ Doped Cs_2_NaScCl_6_: Broadband near-Infrared
Luminescence and Jahn-Teller Effect. J. Chem.
Phys..

[19] Reber C., Güdel H. U. (1988). Near-Infrared
Luminescence Spectroscopy and Relaxation
Behaviour of V^3+^ Doped in Cs_2_NaYCl_6–m_Br_m_(m = 0, 0.3, 3, 6). J. Lumin..

[20] Cao X., Kang L., Guo S., Zhang M., Lin Z., Gao J. (2019). Cs_2_NaVCl_6_: A Pb-Free Halide Double Perovskite
with Strong Visible and near-Infrared Light Absorption. ACS Appl. Mater. Interfaces.

[21] Krimi M., Akermi M., Hassani R., Ben Rhaiem A. (2024). Optical and
Conduction Mechanism Study of Lead-Free CsMnCl_3_ Perovskite. Solid State Sci..

[22] Oetliker U., Güdel H. U. (1991). Optical
Spectroscopic Properties of the Pure Hexagonal
V^3+^-Halide Elpasolites: Cs_2_LiVBr_6_, Cs_2_LiVCl_6_ and Rb_2_LiVCl_6_. J. Lumin..

[23] Ishii Y., Narumi Y., Matsushita Y., Oda M., Kida T., Hagiwara M., Yoshida H. K. (2021). Field-induced successive
phase transitions
in the *J*
_1_–*J*
_2_ buckled honeycomb antiferromagnet Cs_3_Fe_2_Cl_9_. Phys. Rev. B.

[24] Brüning D., Fröhlich T., Gorkov D., Císařová I., Skourski Y., Rossi L., Bryant B., Wiedmann S., Meven M., Ushakov A., Streltsov S. V., Khomskii D., Becker P., Bohatý L., Braden M., Lorenz T. (2021). Multiple field-induced phases in
the frustrated triangular magnet Cs_3_Fe_2_Br_9_. Phys. Rev. B.

[25] Stranger R., Smith P. W., Grey I. E. (1989). Magneto-Structural
Correlations and
Metal-Metal Bonding in Exchange-Coupled Nonahalodimolybdate (A_3_Mo_2_X_9_; X = Chlorine, Bromine, Iodine)
Complexes. Inorg. Chem..

[26] Stranger R., Grey I. E., Madsen I. C., Smith P. W. (1987). Structure Systematics
in A_3_Mo_2_X_9_, X = Cl, Br, I, from Rietveld
Refinement of X-Ray Powder Data. J. Solid State
Chem..

[27] Hill A. H., Harrison A., Dickinson C., Zhou W., Kockelmann W. (2010). Crystallographic
and Magnetic Studies of Mesoporous Eskolaite, Cr_2_O_3_. Microporous Mesoporous Mater..

[28] Bradley A. J., Thewlis J. (1927). The Crystal Structure
of α-Manganese. Proc. R. Soc. London,
Ser. A.

[29] Hull A. W. (1921). X-Ray Crystal
Analysis of Thirteen Common Metals. Phys. Rev..

[30] Hull A. W. (1922). The Crystal
Structures of the Common Elements. J. Franklin
Inst..

[31] Shikano M., Ishiyama O., Inaguma Y., Nakamura T., Itoh M. (1995). Structure
and Magnetic Properties of 6-Layered Hexagonal Oxides Ba_3_Cr_2_MO_9_ (M = Mo and W). J. Solid State Chem..

[32] Kjems, J. K. ; Petitgrand, D. ; Feile, R. ; Leuenberger, B. ; Güdel, H. U. Singlet Ground-State Dimer Systems Cs_3_Cr_2_Br_9_ and Cs_3_Cr_2_Cl_9_ . In Springer Series in Solid-State Sciences; Springer, 1984; Vol. 54, pp 189–194 10.1007/978-3-642-82369-5_34.

[33] Jørgensen, C. K. Electron Transfer Spectra. In Progress in Inorganic Chemistry; John Wiley & Sons, 1970; pp 101–158 10.1002/9780470166130.ch2.

[34] Lever, A. B. P. Inorganic Electronic Spectroscopy, 2nd Ed.; Elsevier: Amsterdam, 1984.

