# Supplementary material for: Metal–Metal Interactions in Hexagonal Perovskites Containing Bimetallic Face-Sharing Bioctahedra
Source: Inorg Chem. 2026 Sep 9;65(37):21819–27. doi: 10.1021/acs.inorgchem.6c03181 (PMC13599384; doi:10.1021/acs.inorgchem.6c03181)
Supplement: Supplementary file 1 [file ic6c03181_si_001.pdf]

Metal-Metal Interactions in Hexagonal Perovskites Containing Bimetallic Face-Sharing Bioctahedra

David Liu[1], Cierra Foster[1], Maxim Avdeev[2,3], and Patrick M. Woodward[1]*

[1] Department of Chemistry and Biochemistry, The Ohio State University, 100 W. 18th Avenue, Columbus, Ohio 43210, United States

[2] Australian Centre for Neutron Scattering, Australian Nuclear Science and Technology Organization, Lucas Heights, NSW 2234, Australia

[3] School of Chemistry, The University of Sydney, NSW 2006, Australia

* Corresponding Author:

PMW: woodward@chemistry.ohio-state.edu

<u>Supplemental Information</u>

Diffraction Space Group Assignment Analysis:

In our analysis of the neutron powder diffraction data of $Cs_3NaMnCrCl_9$, we noticed that the refinements resulted in the cation in the dimer positions relaxing to unreasonable positions. This suggested the neutrons were not sufficiently sensitive to the positions of the Mn and Cr ions. Although initially perplexing, this behavior can be rationalized. Manganese and chromium have neutron scattering lengths (−3.73 fm & 3.635 fm) of similar magnitude but of opposite sign. Hence, if there is a near equivalent mixture of the two ions on a given crystallographic site, it would lead to negligible scattering. For that reason, refinements that use a fixed 50:50 ratio of Mn and Cr in a shared site are unable to accurately resolve its position. This is evident in the $P6_3/mmc$ space group, which assumes a 50:50 ratio due to symmetry and stoichiometry constraints. Refinements with this model lead to cation positions that relaxed to the far ends of the dimers. Not only are these cation positions divergent from positions obtained from Rietveld analysis of synchrotron XRPD data, they lead to chemically unreasonable bond distances.

To confirm this effective cancellation of scattering, Rietveld refinements were carried out on NPD data with a structural model with $P6_3/mmc$ symmetry and the Mn and Cr ions removed from the structure. This approach led to an almost identical fit to the data, R_{wp} = 3.747% without Mn and Cr vs R_{wp} = 3.726% when they are present. Alternatively, refinements in the $P6_3mc$ model, where there are two unique cation sites in the dimer should give an improved fit if cation ordering is present. Instead, we again observe the cations relaxing to positions that do not make chemical sense. This inability to accurately resolve the cation positions would support the presence of cation disorder in the dimers.

To further examine the contrast of Mn and Cr in the neutron refinements, we fixed the atomic coordinates in the $P6_3mc$ model to those determined from XRPD and carried out refinements with varying degrees of cation ordering. When the occupancies were fixed to impose perfect cation order the fit (R_{wp}: 4.461%) was inferior to refinements where a 50:50 mixture of the two ions was imposed on both sites (R_{wp}: 4.282%). If the occupancy was refined the fit improved marginally (R_{wp}: 4.266%) and the occupancies refined to a 0.62(3):0.38(3) ratio. While we cannot rule out low levels of partial Mn/Cr site ordering, taken on the whole analysis of the NPD data strongly supports adoption of the $P6_3/mmc$ space group assignment with no long-range ordering of the Mn and Cr ions.

On the other hand, in $Cs_3NaMnVCl_9$, we focused our analysis on the synchrotron XRPD data. Fitting the synchrotron spectrum with the $P6_3mc$ model does provide hints about the dimer occupancy. The $P6_3mc$ model provides a slightly improved fit over $P6_3/mmc$. It also indicates a dimer arrangement where one octahedron is larger in volume than the other. This would support the inequivalent occupancy of V^{3+} and Mn^{2+} in those sites considering their contrasting ionic radii. The Na^+ cation is also displaced towards the layer of larger volume octahedra (assumed to be Mn rich), which is consistent with the partially ordered 6H variants of $Cs_3NaM^{2+}RhCl_9$. Attempts at refining the dimer cation occupancies led to large uncertainties with values that suggested the smaller octahedron was Mn^{2+} rich, which is inconsistent with ionic radii considerations. We also attempted refinements with the occupancies fixed to 0:1, 0.5:0.5, and 1:0. This resulted in fits that differed by approximately ±0.010% R_{wp}, which further illustrates the poor contrast of X-rays between Mn and V. As such, even though there are structural indicators that support the possibility of cation site ordering, similar features are seen in refinements of the Cr and Fe-analogues when refined in the $P6_3mc$ model, even though they clearly lack site ordering based on neutron analysis. Furthermore, since the Mn-Cr and Mn-Fe pairings have been shown to be disordered, there is no reason to suggest the Mn-V would be any different.

Table S1: Crystallographic information of $Cs_3NaMnCrCl_9$ and $Cs_3NaMnVCl_9$ determined from synchrotron XRPD pattern collected at 300 K.

	$Cs_3NaMnCrCl_9$	$Cs_3NaMnVCl_9$
Crystal System	Hexagonal	Hexagonal
Space Group	$P6_3/mmc$	$P6_3/mmc$
λ (Å)	0.3719	0.2241
a (Å)	7.3456(1)	7.36000(8)
c (Å)	18.1231(4)	18.1510(4)
α (°)	90	90
β (°)	90	90
γ (°)	120	120
V (A^3)	846.87(3)	851.50(3)
Density (g cm^{-3})	3.3244(1)	3.3022(1)
R_{wp} (%)	8.322	3.492

Table S2: Atomic positions of $Cs_3NaMnCrCl_9$ determined from synchrotron XRPD collected at 300 K.

Atom	Wyckoff Site	*x*	*y*	*z*	Occ	B_{eq}
Cs1	2b	0	0	¼	1	2.29(3)
Cs2	4f	1/3	2/3	0.0828(1)	1	2.29(3)
Na1	2a	0	0	0	1	1.1(3)
Mn1	4f	1/3	2/3	0.6592(2)	0.5	0.55(9)
Cr1	4f	1/3	2/3	0.6592(2)	0.5	0.55(9)
Cl1	6h	0.5186(4)	0.0372(8)	¼	1	1.80(6)
Cl2	12k	0.1749(3)	0.3499(6)	−0.0898(2)	1	1.80(6)

Table S3: Atomic positions of $Cs_3NaMnVCl_9$ determined from synchrotron XRPD pattern collected at 300 K.

Atom	Wyckoff Site	*x*	*y*	*z*	Occ	B_{eq}
Cs1	2b	0	0	¼	1	2.05(2)
Cs2	4f	1/3	2/3	0.08372(7)	1	2.05(2)
Na1	2a	0	0	0	1	0.9(2)
Mn1	4f	1/3	2/3	0.6585(1)	0.5	1.03(7)
V1	4f	1/3	2/3	0.6585(1)	0.5	1.03(7)
Cl1	6h	0.5151(3)	0.0301(5)	¼	1	2.02(4)
Cl2	12k	0.1747(2)	0.3493(4)	−0.0888(1)	1	2.02(4)

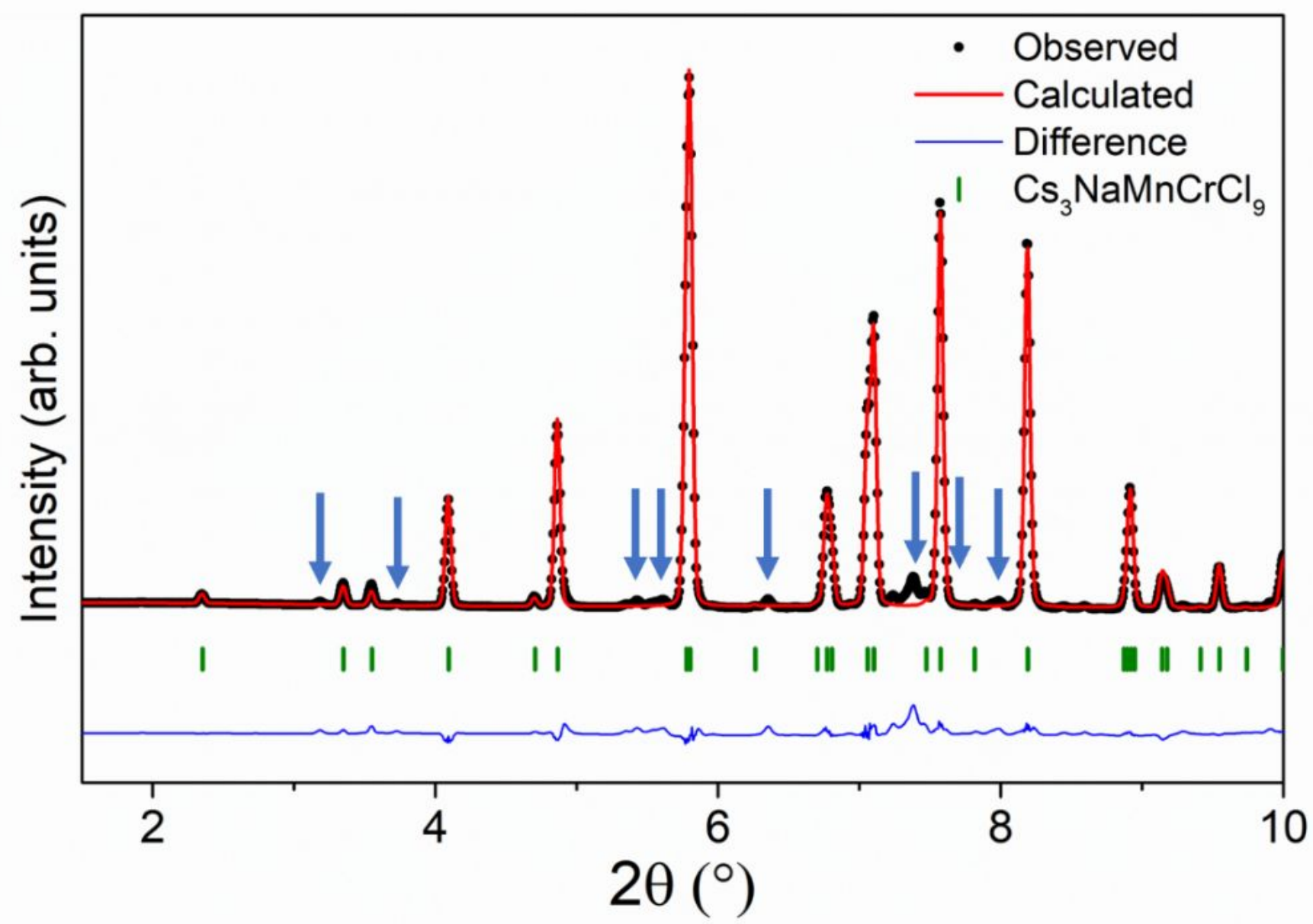

Figure S1: Arrows identifying reflections of an unidentified phase(s) in the synchrotron XRPD of $Cs_3NaMnCrCl_9$.

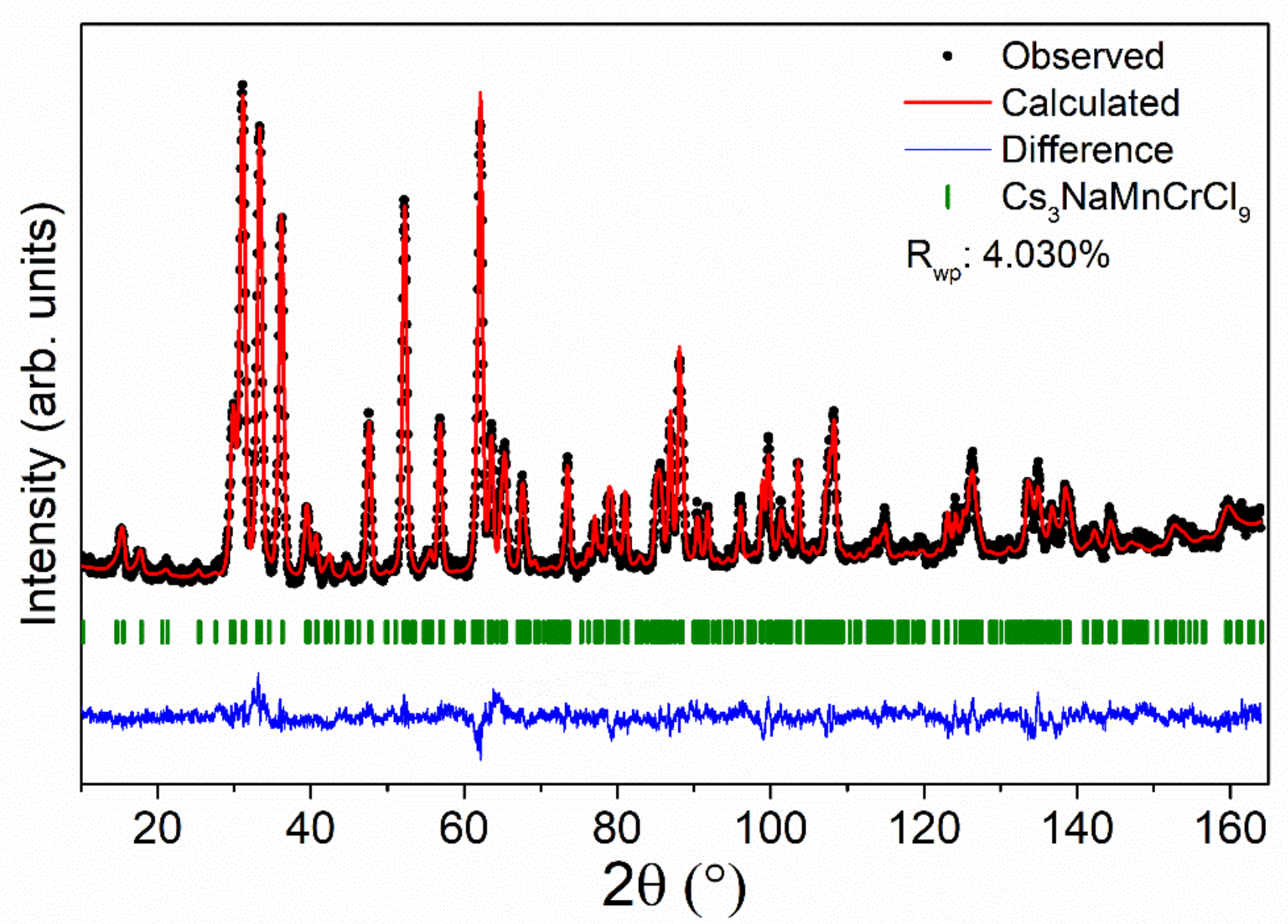

Figure S2: Rietveld refinement of the 300 K neutron powder diffraction pattern of $Cs_3NaMnCrCl_9$ in the $P6_3/mmc$ model with atomic coordinates fixed to those determined from the synchrotron XRPD refinements.

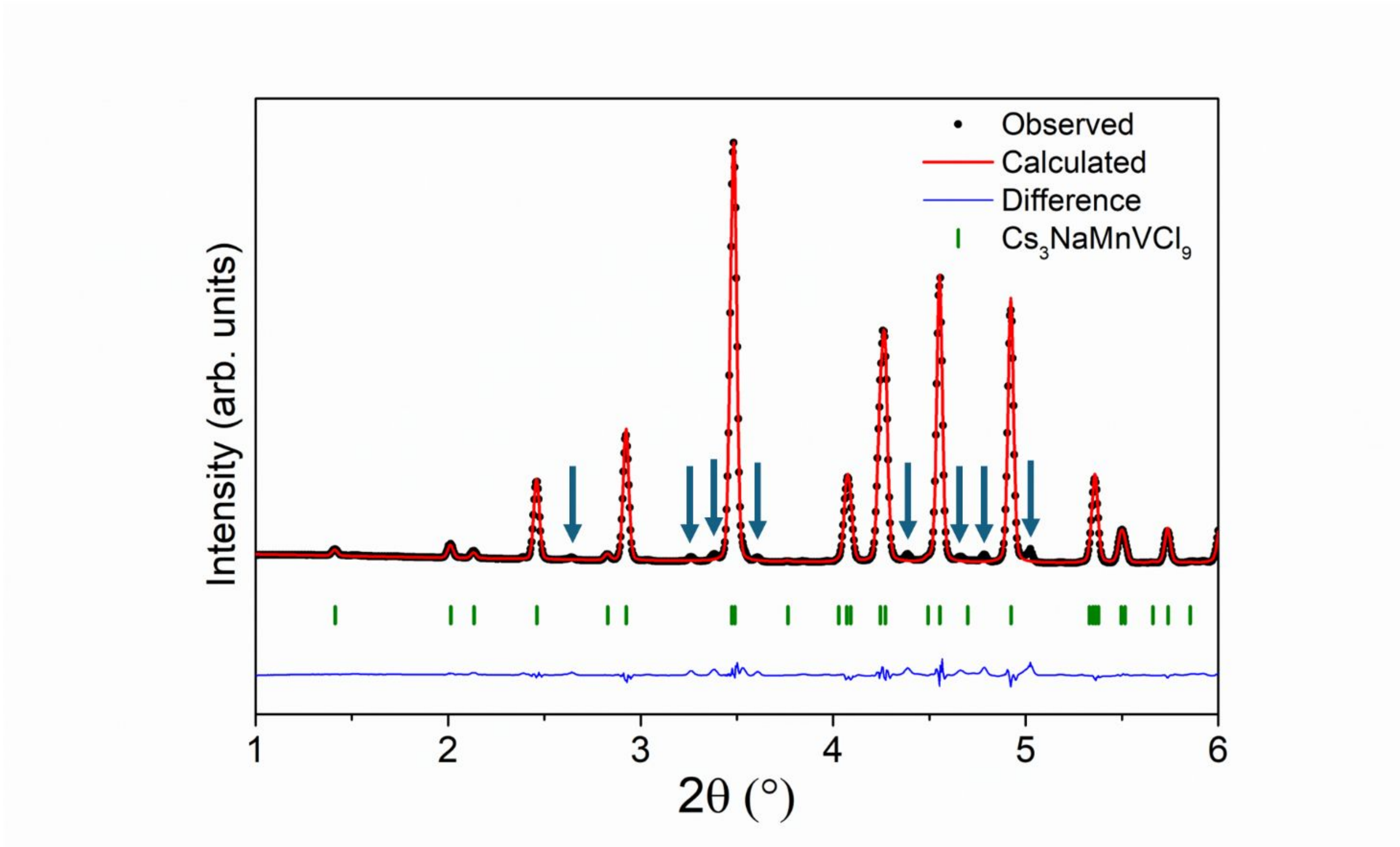

Figure S3: Arrows identifying reflections of an unidentified phase(s) in the synchrotron XRD of $Cs_3NaMnVCl_9$.

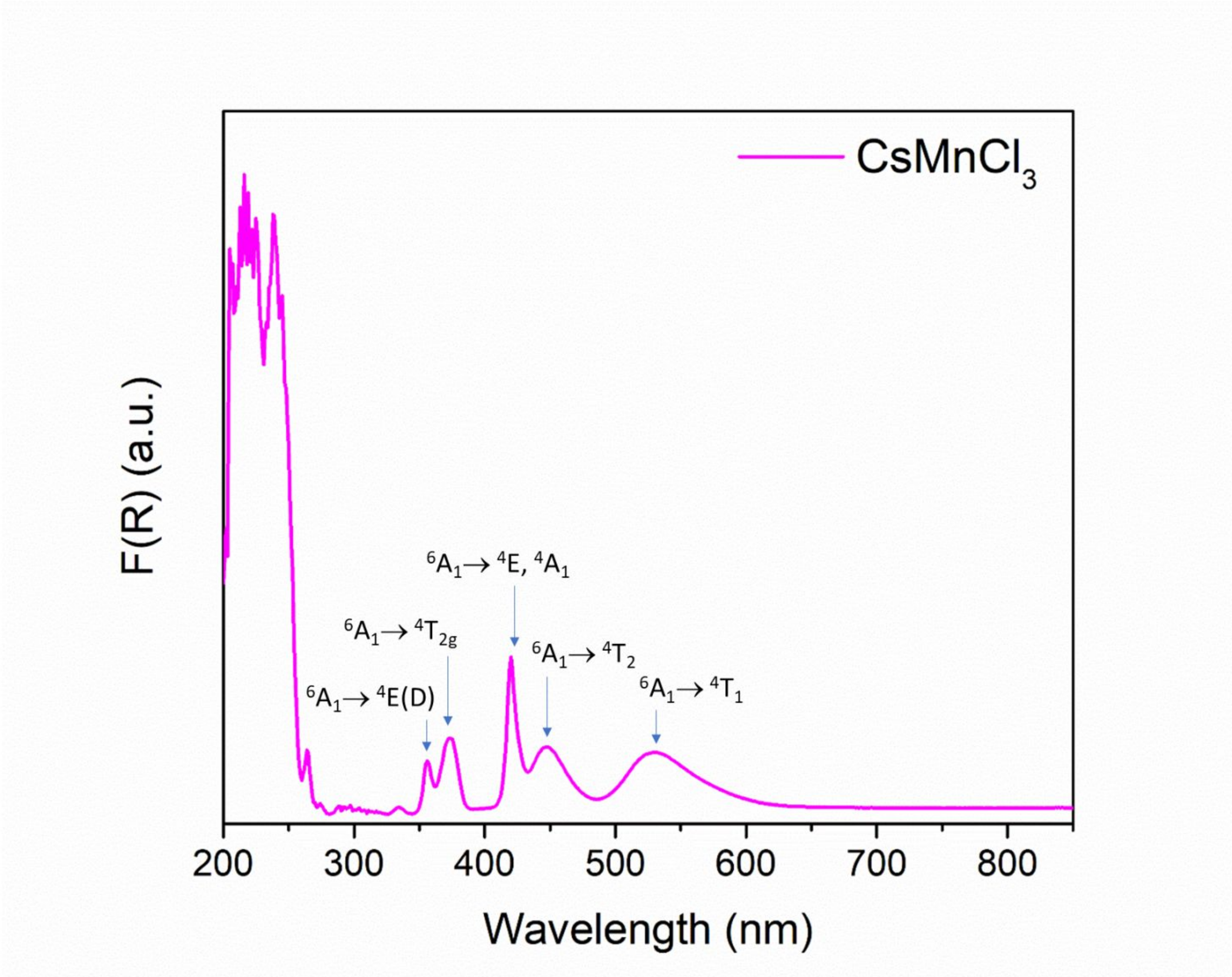

Figure S4: The pseudo-absorbance plot of $CsMnCl_3$.

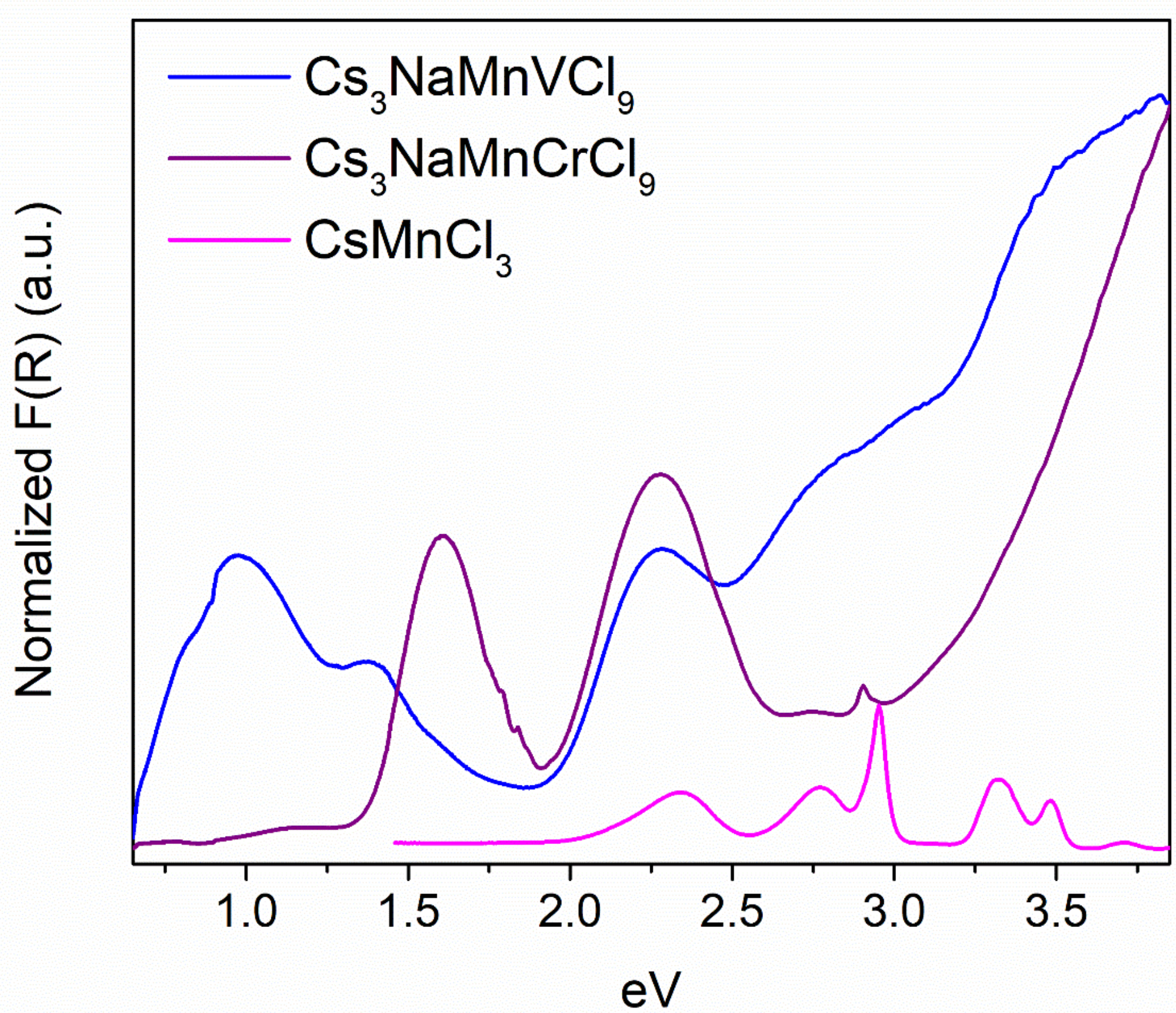

Figure S5: The pseudo-absorbance plot of $CsMnCl_3$, $Cs_3NaMnCrCl_9$, and $Cs_3NaMnVCl_9$.

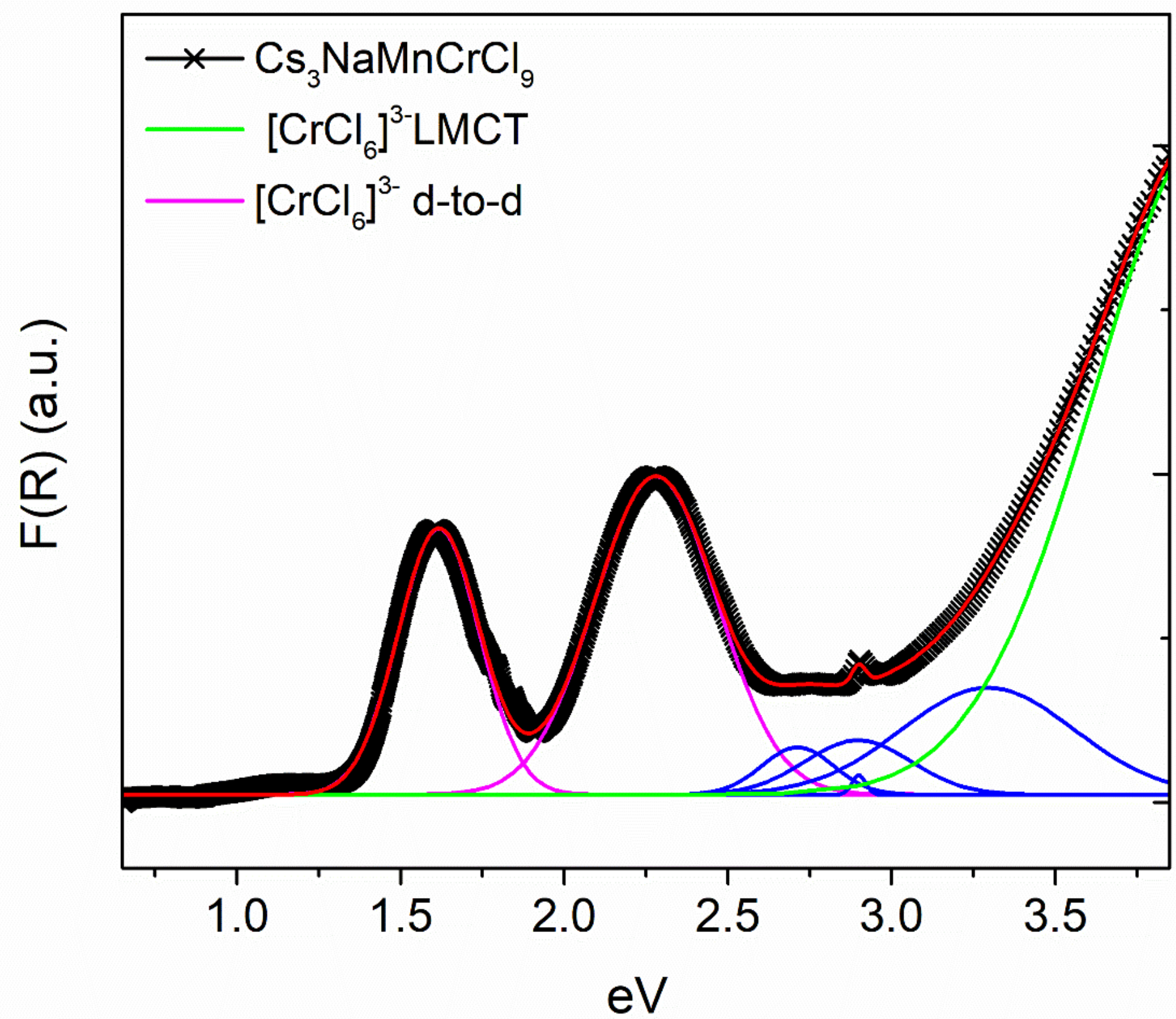

Figure S6: The pseudo-absorbance plot of $Cs_3NaMnCrCl_9$ fitted to gaussian curves.

Table S4: The peak parameters associated with each gaussian curve fitted to the pseudo-absorbance plot of $Cs_3NaMnCrCl_9$,

Sample (Adj. R-Square)	Peak Area	FWHM (eV)	Max Height	Center Gravity (eV)	Center Gravity (nm)
$Cs_3NaMnCrCl_9$ (0.997182)	0.1266(4)	0.294(1)	0.405	1.6172(4)	766.8(2)
	0.229(2)	0.443(4)	0.485	2.280(2)	543.9(5)
	0.02(8)	0.2(2)	0.0720	2.71(5)	458(8)
	0.0016(3)	0.05*	0.0293	2.9*	428
	0.0(2)	0.4(8)	0.0828	2.9(3)	430(40)
	0.1(9)	1(1)	0.163	3.3(5)	380(60)
	1(1)	1(1)	1.03	4.00(5)	310(4)

* denotes parameters fixed during fit.

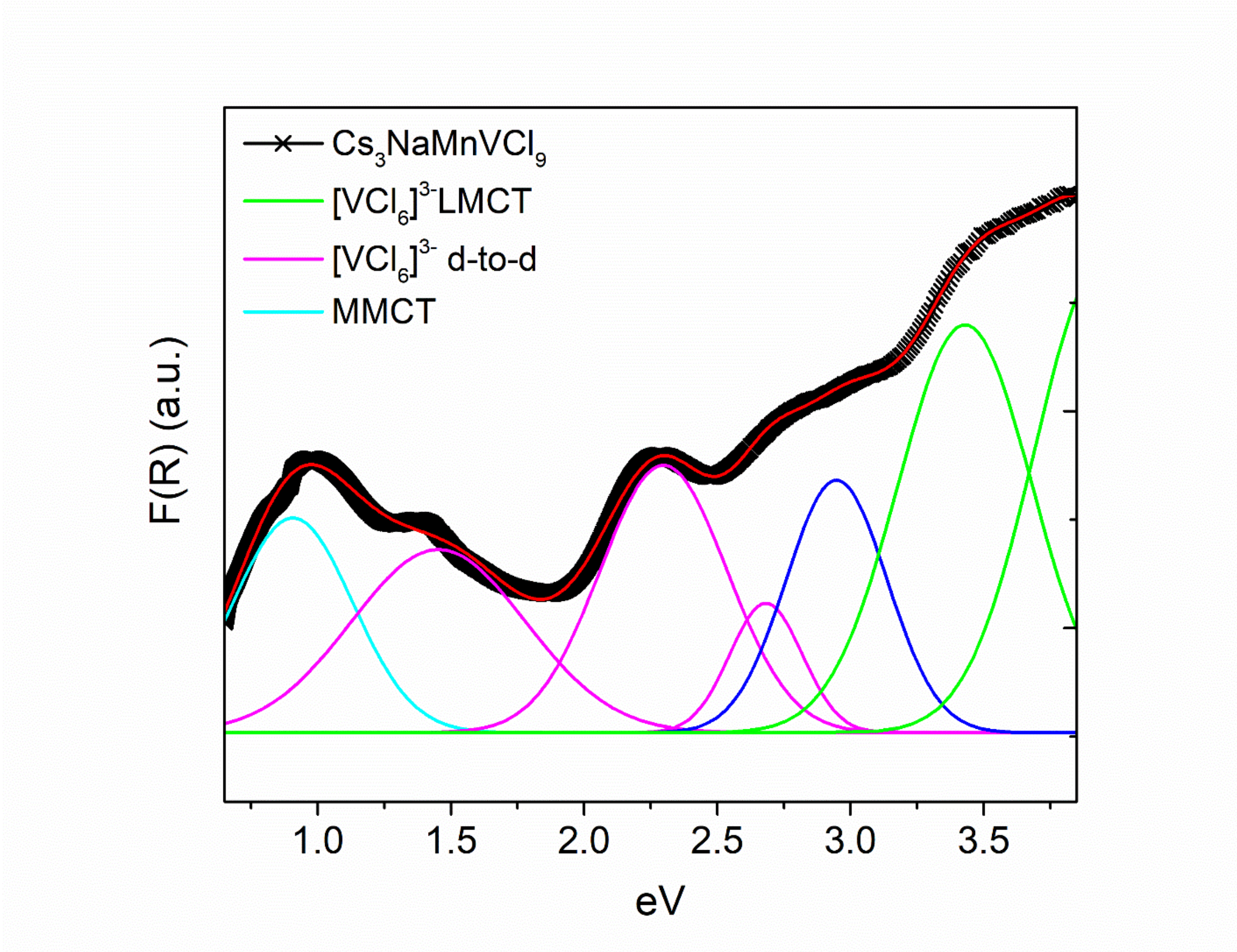

Figure S7: The pseudo-absorbance plot of $Cs_3NaMnVCl_9$ fitted to gaussian curves.

Table S5: The peak parameters associated with each gaussian curve fitted to the pseudo-absorbance plot of $Cs_3NaMnVCl_9$,

Sample (Adj. R-Square)	Peak Area	FWHM (eV)	Max Height	Center Gravity (eV)	Center Gravity (nm)
$Cs_3NaMnVCl_9$ (0.995580)	0.28(2)	0.53(1)	0.495	0.907(4)	1367(6)
	0.35(2)	0.77(3)	0.422	1.45(1)	855(6)
	0.37(2)	0.56(2)	0.617	2.298(4)	539.6(9)
	0.1(1)	0.32(6)	0.299	2.68(4)	463(7)
	0.3(5)	0.4(3)	0.582	2.95(5)	420(7)
	1(1)	0.6(7)	0.940	3.4(3)	360(30)
	1(1)	0.6(7)	1.08	3.9(2)	320(20)
